# Sex-related variability of white matter tracts in the whole HCP cohort

**DOI:** 10.1007/s00429-024-02833-0

**Published:** 2024-07-16

**Authors:** B. Herlin, I. Uszynski, M. Chauvel, S. Dupont, C. Poupon

**Affiliations:** 1 BAOBAB, NeuroSpin, Université Paris-Saclay, CNRS, CEA, Gif-Sur-Yvette, France; 2 Reference Center for Rare Epilepsies, Department of Neurology, Epileptology Unit, AP-HP, Pitié-Salpêtrière Hospital, Paris, France; 3 Rehabilitation Unit, AP-HP, Pitié-Salpêtrière Hospital, Paris, France; 4 Paris Brain Institute (ICM), Sorbonne-Université, Inserm U1127, CNRS 7225 Paris, France; 5 Université Paris Sorbonne, Paris, France

**Keywords:** Diffusion Magnetic Resonance Imaging, White matter, Microstructure, Tractography, Sex differences

## Abstract

Behavioral differences between men and women have been studied extensively, as have differences in brain anatomy. However, most studies have focused on differences in gray matter, while white matter has been much less studied. We conducted a comprehensive study of 77 deep white matter tracts to analyze their volumetric and microstructural variability between men and women in the full Human Connectome Project (HCP) cohort of 1065 healthy individuals aged 22–35 years. We found a significant difference in total brain volume between men and women (+ 12.6% in men), consistent with the literature. 16 tracts showed significant volumetric differences between men and women, one of which stood out due to a larger effect size: the corpus callosum genu, which was larger in women (+ 7.3% in women, p = 5.76 × 10^–19^). In addition, we found several differences in microstructural parameters between men and women, both using standard Diffusion Tensor Imaging (DTI) parameters and more complex microstructural parameters from the Neurite Orientation Dispersion and Density Imaging (NODDI) model, with the tracts showing the greatest differences belonging to motor (cortico-spinal tracts, cortico-cerebellar tracts) or limbic (cingulum, fornix, thalamo-temporal radiations) systems. These microstructural differences may be related to known behavioral differences between the sexes in timed motor performance, aggressiveness/impulsivity, and social cognition.

**Supplementary Information:**

The online version contains supplementary material available at 10.1007/s00429-024-02833-0.

## Introduction

In recent decades, the study of brain differences between men and women has been a topic of controversy. This research stems from and continues the logical progression of cognitive and behavioral studies examining purported sex differences conducted in the latter part of the twentieth century. Following extensive debate, a global consensus has emerged suggesting that men and women function similarly across the vast majority of brain functions, with only a few specific exceptions (Hall 1978; Archer 2019; Hyde 2005; Giudice et al. 2012): men exhibit slightly faster motor responses in time-limited tasks, a greater propensity towards aggressive behavior and violence, and a higher level of sexual interest, whereas women tend to have higher social interests and abilities. The cause of these differences remains under debate, with questions lingering regarding whether they arise from distinct social expectations based on gender, variances in brain anatomy, or a combination of both factors.

Numerous studies have investigated brain anatomical differences between men and women, as summarized in three recent meta-analyses (Jahanshad and Thompson 2017; Eliot et al. 2021; Salminen et al. 2022). One of the primary distinctions is the larger intracranial volume (Ruigrok et al. 2014) and total brain volume (Kaczkurkin et al. 2019) observed in men, a phenomenon evident not only in adults but also in children and adolescents (Kaczkurkin et al. 2019), with a relative difference of 9 to 12% between men and women. This variance in total brain volume necessitates consideration when examining and comparing regional brain volumes. Many initially reported differences, such as a purportedly higher white matter/gray matter ratio in women (Chen et al. 2007; Cosgrove et al. 2007), became either insignificant or of very small effect size after adjusting for brain volume (Leonard et al. 2008; Jäncke et al. 2014; Luders et al. 2009). Extensive research has also been conducted on focal gray matter volume differences between men and women. For instance, an analysis of 200 subjects from the Human Connectome Project (HCP) cohort (Yang et al. 2020) revealed slightly greater cortical thickness in men after correcting for intracranial volume. Moreover, a morphological gray matter analysis of the entire HCP cohort (Luo et al. 2019) demonstrated high sex classification accuracy (96.77%), primarily attributable to differences in frontal areas. While many cortical and subcortical areas exhibited slight differences between men and women, these effects were predominantly small (Eliot et al. 2021 Jun; Ruigrok et al. 2014; Ritchie et al. 2018). Large meta-analyses conducted by the ENIGMA (Enhancing Neuro Imaging Genetics through Meta-Analysis) consortium (Frangou et al. 2021; Dima et al. 2021; Wierenga et al. 2022), encompassing between 16,683 and 18,605 healthy individuals depending on the study, confirmed lower cortical thickness in women in most areas after adjusting for total brain volume. Additionally, these analyses indicated greater volume in men across most subcortical areas, along with increased interindividual variability in men for both cortical and subcortical measures. In summary, gray matter differences between men and women exhibit small effect sizes and are notably less pronounced than interindividual differences.

Unfortunately, there are significantly fewer studies investigating the same question for white matter. One notable exception is the examination of differences in corpus callosum volume between both sexes. This issue emerged from postmortem dissection studies (DeLacoste-Utamsing and Holloway 1982; RichardJ 2005), which indicated a larger corpus callosum in women after adjusting for brain weight or size. Subsequent studies, particularly in larger cohorts facilitated by MRI advancements, have suggested that differences in corpus callosum volume are primarily influenced by the total brain volume (Luders et al. 2014; Pietrasik et al. 2020). Nevertheless, when comparing men and women with identical intracranial volume, the corpus callosum was still found to be larger in women (Ardekani et al. 2013; Shiino et al. 2017).). Ultimately, sex was estimated to account for approximately 1% of the variance in corpus callosum volume (Potvin et al. 2016). Regarding diffusion MRI metrics, such as the simplest ones derived from the diffusion tensor imaging (DTI) model (Basser et al. 1994) like fractional anisotropy (FA) or other metrics derived from more complex models, results generally lack consensus. Depending on the study, FA has been reported to be higher overall in women (Kanaan et al. 2012; Dunst et al. 2014; Cahn et al. 2021), higher overall in men (Ritchie et al. 2018; Cox et al. 2016; Lawrence et al. 2021), or with variable results depending on specific tracts (Kanaan et al. 2012, 2014) or areas (Hsu et al. 2008; Inano et al. 2011; Chou et al. 2011). The ENIGMA consortium also examined white matter (Kochunov et al. 2015) and concluded in its meta-analysis that FA is slightly higher in women overall (relative difference: + 2%). However, comparing studies is challenging due to methodological differences: some have examined FA and other diffusion metrics globally, across the entire white matter; others have investigated these parameters regionally (e.g., frontal white matter); and still others, particularly the more recent ones, have analyzed these parameters along reconstructed white matter tracts. Moreover, cohorts vary in terms of age and size, with age being a critical factor in such studies as white matter microstructural parameters tend to develop differently in men and women during childhood/adolescence (Lawrence et al. 2023), adulthood (Toschi et al. 2020), and aging (Lawrence et al. 2021). Cohort size is also pivotal in this context, as highlighted in a dedicated meta-analysis (Ruigrok et al. 2014): the influence of sex on white matter is minor, and there is no clear dichotomy between men and women in white matter tracts, but rather an overlap between the two groups. Hence, large cohorts are mandatory to robustly detect these differences, potentially explaining why studies with smaller cohorts have reached differing conclusions.

Therefore, it is of great importance to further investigate the structural differences in brain connectivity between men and women from a large homogeneous cohort. In this study, we systematically compared all deep white matter tracts between the sexes in a large cohort of healthy young adults: the Human Connectome Project (HCP) cohort, which comprises 1065 individuals aged 22 to 35 years old. After conducting whole-brain tractography and atlas-based extraction of deep white matter tracts in all subjects, we conducted a volumetric analysis of each tract normalized to the subject's total brain volume. Additionally, we analyzed microstructural parameters derived from Diffusion Tensor Imaging (DTI), Q-ball Imaging (QBI), and Neurite Orientation Dispersion and Density Imaging (NODDI) models. This approach allowed us to provide a comprehensive overview of both morphological and microstructural differences in deep white matter tracts between the sexes in young healthy individuals.

## Methods

### Database

We used the brain MRI dataset from the Human Connectome Project (Q1-Q4 release, 2015) acquired by Washington University in Saint Louis and the University of Minnesota (Essen et al. 2013). This database includes 1065 healthy individuals aged 22 to 35 years old, 490 men and 575 women with a similar age distribution, all of whom underwent an anatomical T1-weighted (T1w) scan and series of diffusion MRI (dMRI) scans on a Connectome Skyra 3 T MRI scanner. The T1w acquisition was performed using a 3D MPRAGE sequence, with a 0.7 mm isotropic spatial resolution and TR/TE = 2400/2.14 ms. The dMRI acquisitions were performed with a 2D monopolar pulsed gradient spin-echo (PGSE) single-shot multi-band EPI sequence with a multi-band factor of 3, a 1.25 mm isotropic spatial resolution, TR/TE = 5520/ 89.50 ms, and a multiple-shell sampling of the q-space based on 3 b-values of 1000, 2000, and 3000 s/mm2 along 90 uniformly distributed diffusion directions per shell, plus 6 non-diffusion-weighted b = 0 s/mm^2^ reference images. The dataset was already pre-processed and corrected for eddy current and susceptibility artifacts and the dMRI scans of each subject were already aligned to the corresponding T1w scan.

### Individual analysis pipeline

For all subjects, brain parcellation and volumetric segmentation were performed from the anatomical T1-weighted MRI, using the Freesurfer image analysis suite, documented and freely available for download online (http://surfer.nmr.mgh.harvard.edu/).

To process the dMRI data, we designed an analysis pipeline based on the Ginkgo toolbox developed by the CEA/NeuroSpin team and freely available online at https://framagit.org/cpoupon/gkg, which performed four sequential steps for each subject. A global overview of this diffusion analysis pipeline is provided in the Supplementary Material (Fig. S1).

1. dMRI processing by computing the Diffusion Tensor Imaging (DTI) model (Basser et al. 1994) and the Orientation Distribution Functions (ODF) for each voxel of the brain using the analytical Q-ball model (Descoteaux et al. 2007) within constant solid angle (Aganj et al. 2010). These models also provided several quantitative diffusion metrics, such as mean, axial, and radial diffusivities, as well as generalized fractional anisotropy, which was used to regularize fiber trajectories (Perrin et al. 2005). The ODF maps were computed using all 3 shells, and the quantitative DTI metrics were computed using only the b = 1000 s/mm^2^ shell. We also computed the Neurite Orientation Dispersion and Density Imaging (NODDI) model (Zhang et al. 2012) from all 3 shells, which allows the estimation of additional microstructural parameters.
2. Computation of a whole-brain tractogram from the ODF map using a regularized probabilistic algorithm (Perrin et al. 2005) (parameters: 8 seeds per voxel over a predefined propagation domain computed from the T1w image, aperture angle of 30°, fiber length range of 1.25—300 mm, forward and backward integration step of 0.3 mm, Gibb’s sampler temperature of 1). The fiber length range allowed us to discard some artifactuals streamlines (too short streamlines or infinite loops).
3. Registration of the deep white matter atlas into native space, using the subject’s anatomical T1-weighted acquisition and the MNI (Montreal Neurological Institute) ICBM 2009c nonlinear asymmetric template as a reference template. Registration was performed using the Advanced Normalization Tools (ANTs) toolbox, with a diffeomorphic transformation based on the Symmetric Normalization (SyN) approach (Avants et al. 2008, 2011), which computes both the subject-to-MNI and the MNI-to-subject transform. The MNI-to-subject transform is then used to register the deep white matter atlas, located in the MNI space, to the subject space.
4. Automatic bundle segmentation from each tractogram based on a predefined deep white matter atlas (Herlin et al. 2023; Chauvel et al. 2023), using a maximum pairwise distance threshold algorithm between streamlines from the tractogram and labeled white matter bundles from the atlas. This atlas contains 77 tracts (see Table 1): 15 association tracts for each hemisphere, 19 projection tracts for each hemisphere, 8 interhemispheric tracts, and 1 intracerebellar tract. Additional information on the atlas construction is also provided in Supplementary Material, along with an overview of this atlas (supplementary Figs. 1 and 2). Automatic bundle segmentation is performed in the subject space from the the subject's tractogram, after transformation of the deep white matter atlas (expressed in the MNI space) into the subject's space using the inverse diffeomorphic transformation calculated between the T1-weighted MRI and the MNI template. The fiber labeling algorithm iterates over streamlines and computes the minimum pairwise distance between each streamline and the centroid of each white matter tract in the atlas. For a streamline to be assigned to a tract, its distance must be below a predetermined threshold. If a streamline is below the distance threshold of two (or more) different tracts, it is assigned to the one it is the closest to. A streamline can only be assigned to a single tract, thereby eliminating any potential redundancy in adjacent tracts.

**Table 1:** Deep white matter fiber atlas established using the whole HCP cohort

Association fiber bundles (bilateral: 2 × 15 tracts)	Projection fiber bundles (bilateral: 2 × 19 tracts)	Interhemispheric fiber bundles (8 tracts)	Other (1 tract)
Arcuate fasciculus	Fornix	Anterior commissure	Parallel fibers of the cerebellum
Cortico-spinal tract (CST)	Centro-caudate tract	Corpus callosum: anterior midbody	
Cingulum (long)	Cingulo-caudate tract	Corpus callosum: genu	
Dorsal cingulum	Fronto-caudate tract	Corpus callosum: isthmus	
Ventral cingulum	Parieto-caudate tract	Corpus callosum: posterior midbody	
External capsule	Centro-lenticular tract	Corpus callosum: rostral midbody	
Extreme capsule	Fronto-lenticular tract	Corpus callosum: rostrum	
Frontal aslant	Occipito-lenticular tract	Corpus callosum: splenium	
Inferior fronto-occipital fasciculus (IFOF)	Parieto-lenticular tract		
Inferior longitudinal fasciculus (ILF)	Temporo-lenticular tract		
Middle longitudinal fasciculus (MLF)	Inferior cerebellar peduncle: spino-cerebellar tract		
Superior longitudinal fasciculus 1 (SLF1)	Middle cerebellar peduncle: cortico-cerebellar tract		
Superior longitudinal fasciculus 2 (SLF2)	Superior cerebellar peduncle: cortico-cerebellar tract		
Superior longitudinal fasciculus 3 (SLF3)	Optic radiations		
Uncinate fasciculus	Thalamo-central radiations		
	Thalamo-frontal radiations		
	Thalamo-occipital radiations		
	Thalamo-parietal radiations		
	Thalamo-temporal radiations		

### Statistical analysis

Total brain volume (TBV) and white matter volume (WMV) were established from the Freesurfer brain mask. Each white matter tract volume was measured in the subject space by computing the density mask of each bundle and measuring the volume of this mask with a minimum threshold of 5 fibers/voxel. All white matter tract volumes were then normalized to the respective subject’s TBV and expressed as a percentage of the TBV. In addition, all white matter tract volumes were also normalized to the subject’s white matter volume.

The following microstructural parameters were computed for each voxel of the brain, creating a 3D quantitative map for each parameter: fractional anisotropy FA, mean diffusivity MD, axial diffusivity and radial diffusivity (from the DTI model, using only the b = 1000 s/mm^2^ shell for the computation of these quantitative parameters); generalized fractional anisotropy GFA (from the Q-ball model); neurite density index NDI, isotropic water volume fraction IWVF, and orientation dispersion index ODI (from the NODDI model). For each white matter tract and each quantitative map, we computed the mean of the values (after testing for normality using Shapiro–Wilk test) obtained by trilinear interpolation of the quantitative map at all fiber points, resampled to 0.1 mm.

Statistical analyses for group comparisons were performed using Student’s t-test after testing for normality using Shapiro–Wilk test and for homogeneity of variance using Levene’s test. Correction for multiple comparisons was performed using the Bonferroni correction: starting from p = 0.05, after correction for 772 comparisons, the significance threshold was p = 0.000064767 (6.4767.10^–5^).

The effect size was estimated using Cohen's d test. The following ranges were used for its interpretation: |d|< 0.2: negligible effect size; 0.2 <|d|< 0.5: small effect size, 0.5 <|d|< 0.8: medium effect size; |d|> 0.8: large effect size.

To examine the relationship between sex, normalized corpus callosum tract volume and total brain volume, we conducted a linear regression between normalized corpus callosum volume and total brain volume in men and women, as described by (Leonard et al. 2008), using least squares method. Further analyses of the interaction of normalized tract volume, total brain volume, and sex were performed using analysis of covariance (ANCOVA) with the Ordinary Least Squares (OLS) model.

## Results

### Volumetric comparisons

**Fig. 1:**
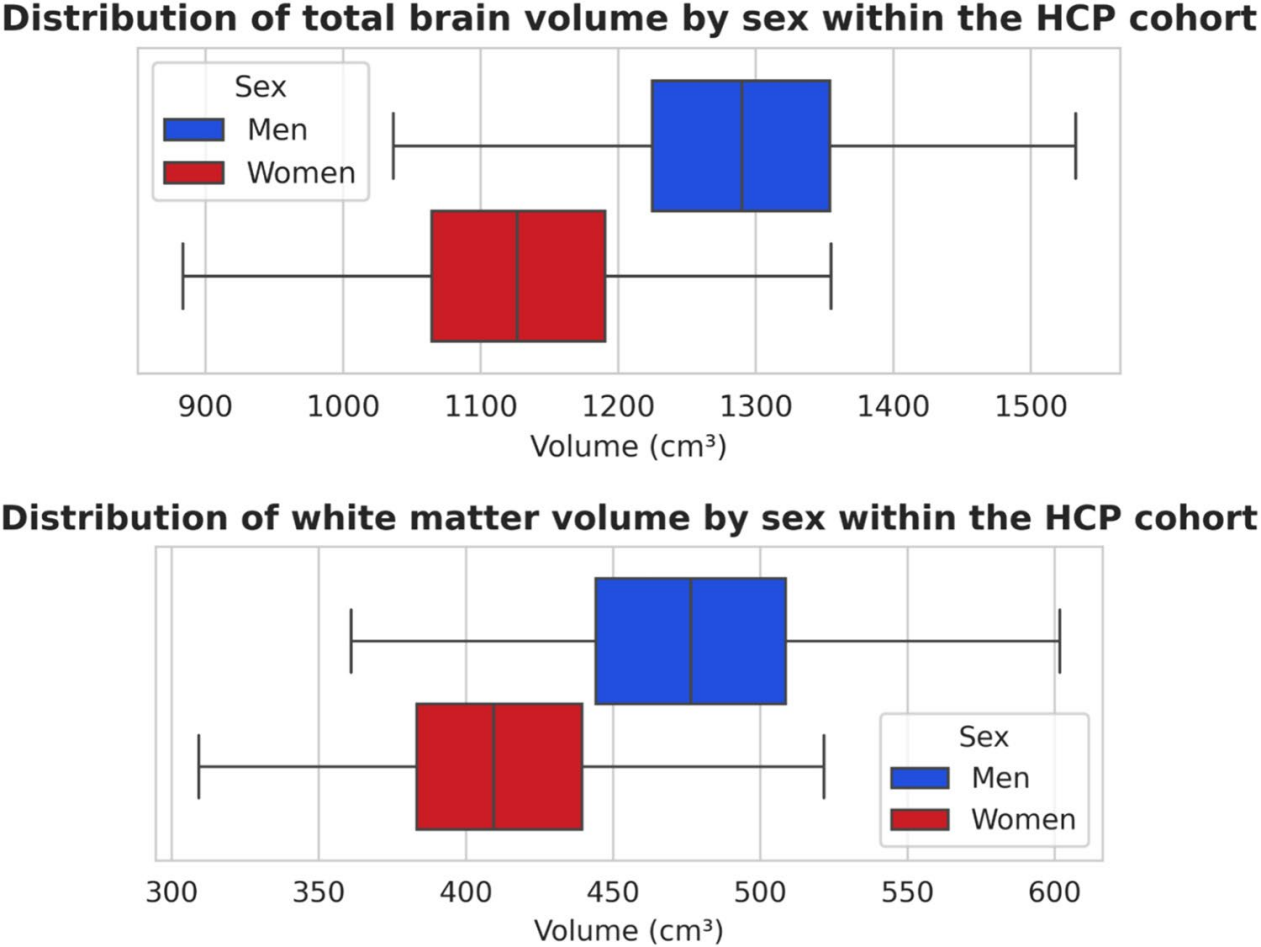
Distribution of total brain volume and white matter volume according to sex within the HCP cohort

Total brain volume was significantly different between men and women (Fig. 1), with a mean ± standard deviation of 1128 ± 90 cm^3^ in women and 1290 ± 102 cm^3^ in men, i.e. a mean relative difference of 12.6% between men and women (p = 1.3 × 10^–127^). The effect size was large (d = 1.7).

White matter volume was also significantly different between men and women (Fig. 1), with a mean ± standard deviation of 409 ± 42 cm^3^ in women and 476 ± 49 cm^3^ in men, i.e. a mean relative difference of 13.6% between men and women (p = 4.0 × 10^–97^). The effect size was large (d = 1.4).

**Fig. 2:**
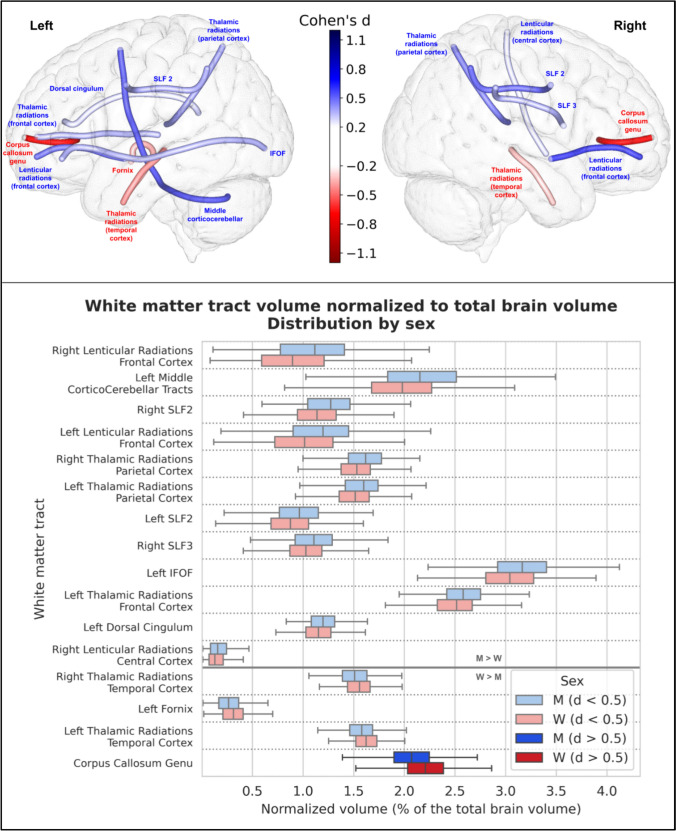
Representation of the 16 white matter tracts with a significant difference in volume normalized to total brain volume. All of these tracts showed a significant difference in their normalized volume between men and women after Bonferroni correction (p < 6.4767.10^–5^). Top: The 16 white matter tracts are represented by their centroid superimposed on a 3D mesh of the brain surface. Left hemisphere tracts are shown on the left side, right hemisphere tracts are shown on the right side, and interhemispheric tracts are shown on both sides. The color represents the direction of the difference (red: greater in women, blue: greater in men), and the color intensity is proportional to the effect size measured by Cohen’s d. Bottom: Distribution of volume normalized to total brain volume of the 16 white matter tracts with a significant difference between men and women, ranked by their Cohen’s d value. 15 tracts showed a small effect size (0.2 < d < 0.5), in light blue (when larger in men) or light red (when larger in women), and only one (the corpus callosum genu bundle) showed a medium effect size (0.5 < d < 0.8), larger in women, in deep red.

16 of the 77 white matter tracts showed a significant difference between men and women in their volume normalized to the total brain brain volume. These results are summarized in Table 2 and Fig. 2, where only the 16 statistically different tracts are shown. The relative difference and Cohen’s d are negative when the volume is greater in women and positive when it is greater in men. Among these significantly different tracts, only one had a Cohen’s d effect size greater than 0.5, i.e., a medium effect size: the corpus callosum genu bundle, which had a greater relative volume in women (7.3%); all the other 15 tracts had a small effect size with a Cohen’s d between 0.2 and 0.5.

**Table 2:** Detailed values of total brain volume and the 16 white matter tracts with a significant difference in volume normalized to total brain volume between men and women, ranked by their Cohen’s d value in descending order. A positive relative difference and Cohen's d value indicates greater volume in men, and a negative relative difference and Cohen’s d value indicates greater volume in women

Tract	Mean (± Std) normalized volume in men (percentage of the TBV)	Mean (± Std) normalized volume in women (percentage of the TBV)	Relative difference (percentage; positive: men > women; negative: women > men)	P-value	Cohen's d (positive: men > women; negative: women > men)
Total Brain Volume	1290 ± 101 cm^3^	1127 ± 90 cm^3^	12.62%	1.3.10^–127^	1.7
Right Lenticular Radiations Frontal Cortex	1.10% (± 0.42)	0.90% (± 0.41)	17.9%	1.72.10^–14^	0.48
Left Middle Cortico-cerebellar Tracts	2.17% (± 0.46)	1.97% (± 0.46)	9.2%	3.72.10^–12^	0.43
Right SLF2	1.26% (± 0.30)	1.14% (± 0.28)	9.8%	8.48.10^–12^	0.42
Left Lenticular Radiations Frontal Cortex	1.17% (± 0.39)	1.01% (± 0.38)	13.3%	7.34.10^–11^	0.4
Right Thalamic Radiations Parietal Cortex	1.61% (± 0.25)	1.51% (± 0.23)	5.7%	5.59.10^–10^	0.38
Left Thalamic Radiations Parietal Cortex	1.58% (± 0.26)	1.50% (± 0.23)	5.1%	1.19.10^–07^	0.33
Left SLF2	0.96% (± 0.28)	0.88% (± 0.27)	9.1%	2.73.10^–07^	0.32
Right SLF3	1.11% (± 0.27)	1.03% (± 0.24)	7.1%	7.20.10^–07^	0.31
Left IFOF	3.15% (± 0.41)	3.04% (± 0.35)	3.7%	1.04.10^–06^	0.3
Left Thalamic Radiations Frontal Cortex	2.58% (± 0.26)	2.50% (± 0.26)	3.0%	1.19.10^–06^	0.3
Left Dorsal Cingulum	1.20% (± 0.17)	1.16% (± 0.17)	3.6%	5.96.10^–05^	0.25
Right Lenticular Radiations Central Cortex	0.17% (± 0.11)	0.15% (± 0.10)	14.9%	5.94.10^–05^	0.25
Right Thalamic Radiations Temporal Cortex	1.50% (± 0.18)	1.55% (± 0.16)	−3.2%	5.66.10^–06^	−0.28
Left Fornix	0.27% (± 0.14)	0.31% (± 0.14)	−15.4%	1.31.10^–06^	−0.3
Left Thalamic Radiations Temporal Cortex	1.57% (± 0.17)	1.63% (± 0.15)	−3.6%	1.32.10^–08^	−0.35
Corpus Callosum Genu	2.06% (± 0.27)	2.21% (± 0.27)	−7.3%	5.76.10^–19^	−0.56

**Fig. 3:**
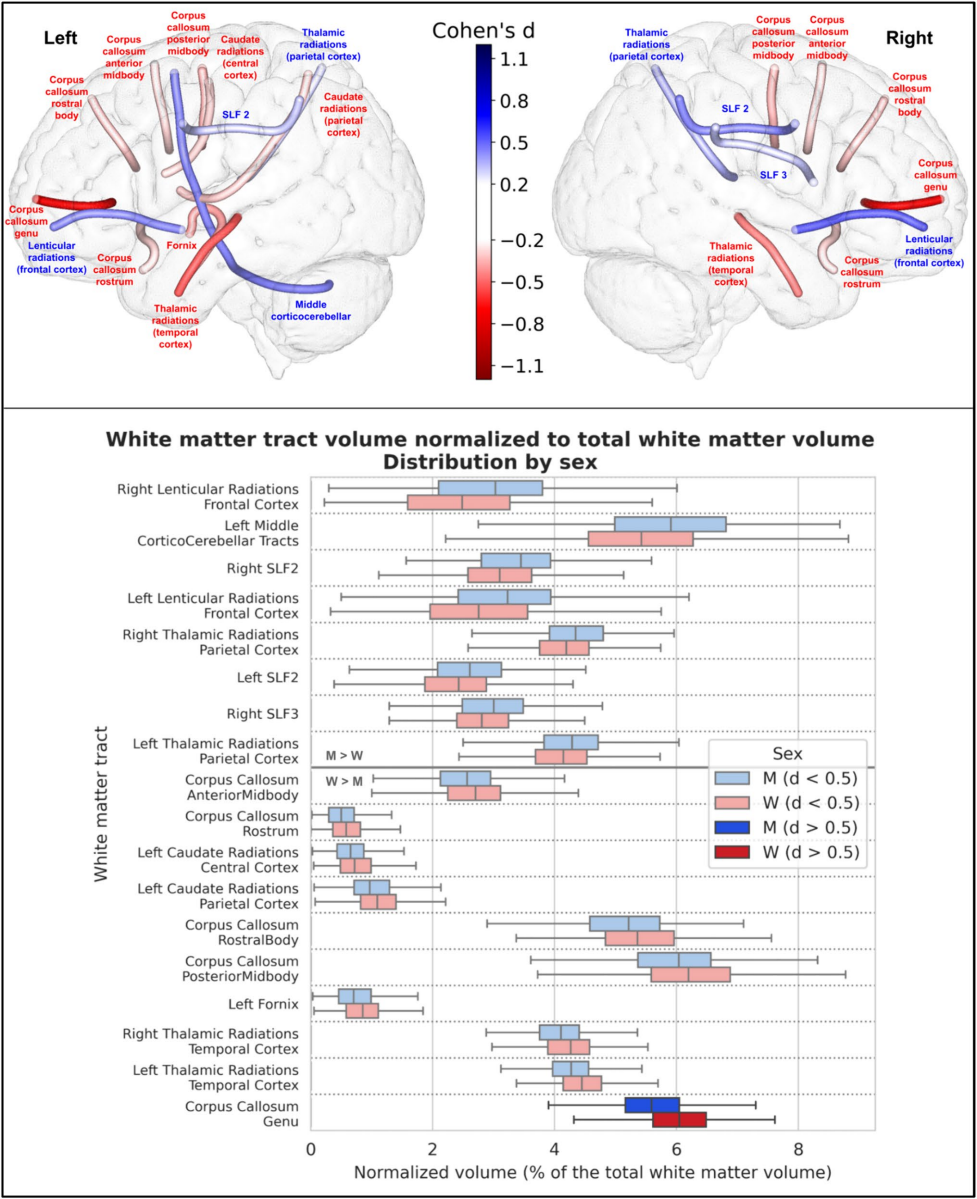
Representation of the 18 white matter tracts with a significant difference in volume normalized to white matter. All of these tracts showed a significant difference in their normalized volume between men and women after Bonferroni correction (p < 6.4767.10^–5^). Top: The 18 white matter tracts are represented by their centroid superimposed on a 3D mesh of the brain surface. Left hemisphere tracts are shown on the left side, right hemisphere tracts are shown on the right side, and interhemispheric tracts are shown on both sides. The color represents the direction of the difference (red: greater in women, blue: greater in men), and the color intensity is proportional to the effect size measured by Cohen’s d. Bottom: Distribution of volume normalized to total brain volume of the 18 white matter tracts with a significant difference between men and women, ranked by their Cohen’s d value. 17 tracts showed a small effect size (0.2 < d < 0.5), in light blue (when larger in men) or light red (when larger in women), and only one (the corpus callosum genu bundle) showed a medium effect size (0.5 < d < 0.8), larger in women, in deep red

We then performed the same analysis after normalizing the individual tract volumes to the individual white matter volume, instead of the individual total brain volume. This yielded similar results: 18 of the 77 white matter tracts also showed a significant difference in white matter-normalized volume between men and women. These results are summarized in Table 3 and Fig. 3, where only the 18 statistically different tracts are shown. As with the tract volumes normalized to total brain volume, only one had a Cohen’s d effect size greater than 0.5, i.e., a medium effect size: the corpus callosum genu bundle, which had a greater relative volume in women (8.5%).

**Table 3:** Detailed values of total white matter volume and the 18 white matter tracts with a significant difference in volume normalized to white matter volume between men and women, ranked by their Cohen’s d value in descending order. A positive relative difference and Cohen’s d value indicates greater volume in men, and a negative relative difference and Cohen’s d value indicates greater volume in women

Tract	Mean (± Std) normalized volume in men (percentage of the WMV)	Mean (± Std) normalized volume in women (percentage of the WMV)	Relative difference (percentage)	P-value	Cohen’s d
Total White Matter Volume	476 ± 49 cm^3^	409 ± 42 cm^3^	13.7%	4.05.10^–97^	1.4
Right Lenticular Radiations Frontal Cortex	2.98% (± 1.13)	2.48% (± 1.12)	16.8%	8.89.10^–13^	0.44
Left Middle Cortico-cerebellar Tracts	5.88% (± 1.27)	5.40% (± 1.26)	8.1%	1.28.10^–09^	0.38
Right SLF2	3.42% (± 0.80)	3.12% (± 0.76)	8.7%	7.82.10^–10^	0.38
Left Lenticular Radiations Frontal Cortex	3.16% (± 1.05)	2.77% (± 1.05)	12.4%	1.96.10^–09^	0.37
Right Thalamic Radiations Parietal Cortex	4.35% (± 0.67)	4.15% (± 0.62)	4.6%	6.05.10^–07^	0.31
Left SLF2	2.61% (± 0.74)	2.40% (± 0.74)	8.0%	4.33.10^–06^	0.28
Right SLF3	2.99% (± 0.71)	2.81% (± 0.64)	6.0%	1.60.10^–05^	0.27
Left Thalamic Radiations Parietal Cortex	4.28% (± 0.70)	4.11% (± 0.64)	3.9%	4.38.10^–05^	0.25
Corpus Callosum Anterior Midbody	2.54% (± 0.58)	2.69% (± 0.62)	−5.9%	5.27.10^–05^	−0.25
Corpus Callosum Rostrum	0.52% (± 0.29)	0.60% (± 0.31)	−14.5%	4.17.10^–05^	−0.25
Left Caudate Radiations Central Cortex	0.67% (± 0.32)	0.76% (± 0.37)	−13.0%	5.95.10^–05^	−0.25
Left Caudate Radiations Parietal Cortex	1.02% (± 0.45)	1.13% (± 0.45)	−11.6%	1.99.10^–05^	−0.26
Corpus Callosum Rostral Body	5.13% (± 0.88)	5.36% (± 0.88)	−4.6%	1.64.10^–05^	−0.27
Corpus Callosum Posterior Midbody	5.93% (± 0.95)	6.21% (± 0.96)	−4.7%	2.05.10^–06^	−0.29
Left Fornix	0.73% (± 0.38)	0.86% (± 0.38)	−16.8%	1.24.10^–07^	−0.33
Right Thalamic Radiations Temporal Cortex	4.07% (± 0.49)	4.25% (± 0.46)	−4.4%	1.05.10^–09^	−0.38
Left Thalamic Radiations Temporal Cortex	4.26% (± 0.47)	4.46% (± 0.45)	−4.8%	5.69.10^–13^	−0.45
Corpus Callosum Genu	5.57% (± 0.70)	6.05% (± 0.70)	−8.5%	1.08.10^–26^	−0.68

**Fig. 4:**
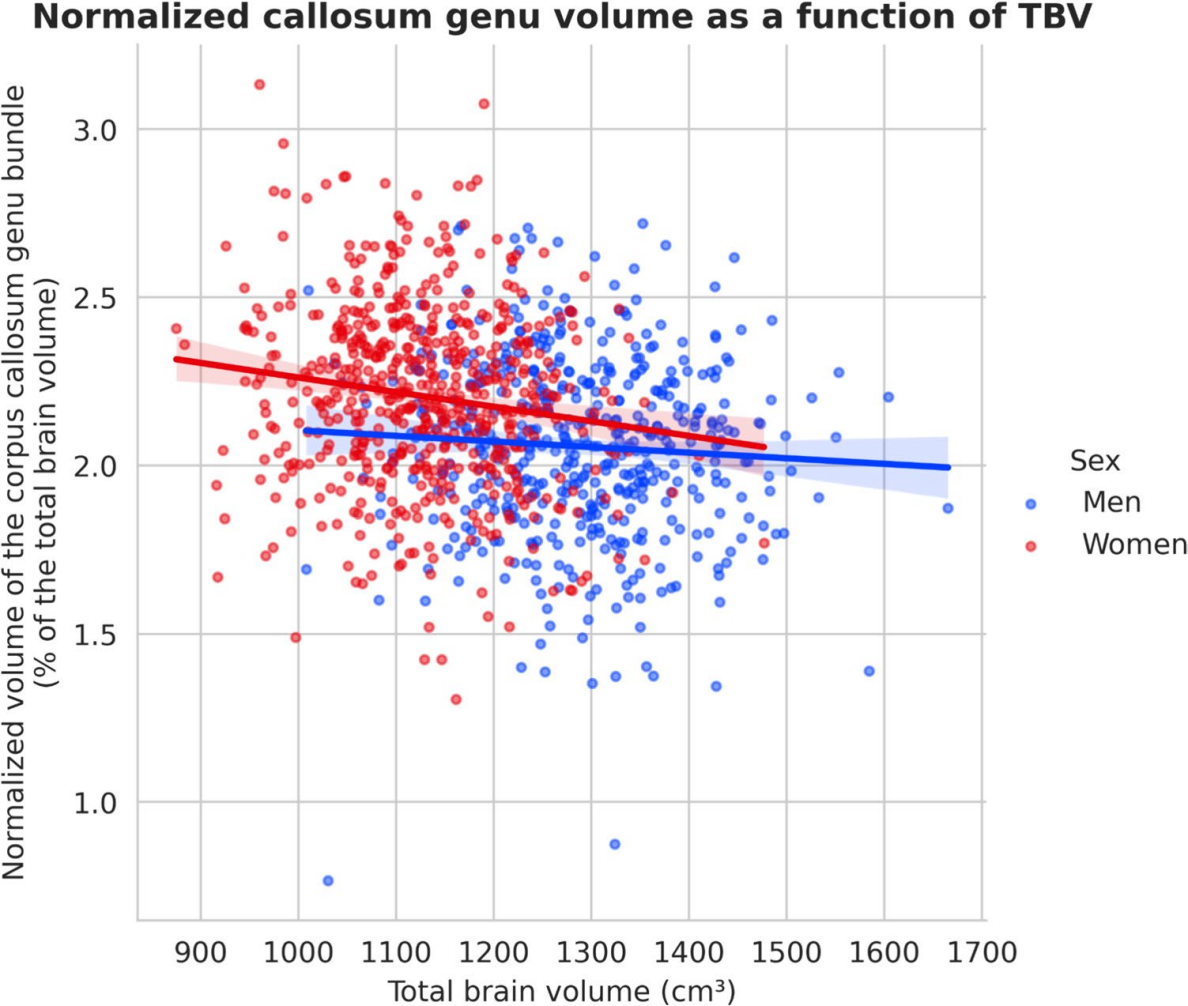
Linear regression of the normalized volume of the corpus callosum genu bundle as a function of the total brain volume

To further examine the relationship between the normalized volume of the most statistically significant tracts (the corpus callosum genu), total brain volume, and sex, and to rule out the possibility that this difference between the sexes was simply a function of total brain volume, we performed a linear regression of the normalized volume of these tracts as a function of the total brain volume. The results are shown in Fig. 4, which shows different slopes between men and women. We then performed an ANCOVA analysis with the normalized volume of the corpus callosum genu as the dependent variable, total brain volume as the continuous variable, and sex as the categorical variable. This revealed a statistically significant interaction between normalized volume of the corpus callosum genu and sex, even when the total brain volume was taken into account (F-statistic = 32.47, p-value = 4.41 × 10^–20^).

### Microstructural comparisons

We performed comparisons between men and women for the various microstructural parameters computed from the DTI, QBI and NODDI models for all tracts. The complete results of these comparisons are shown in Supplementary Table 1.

**Fig. 5:**
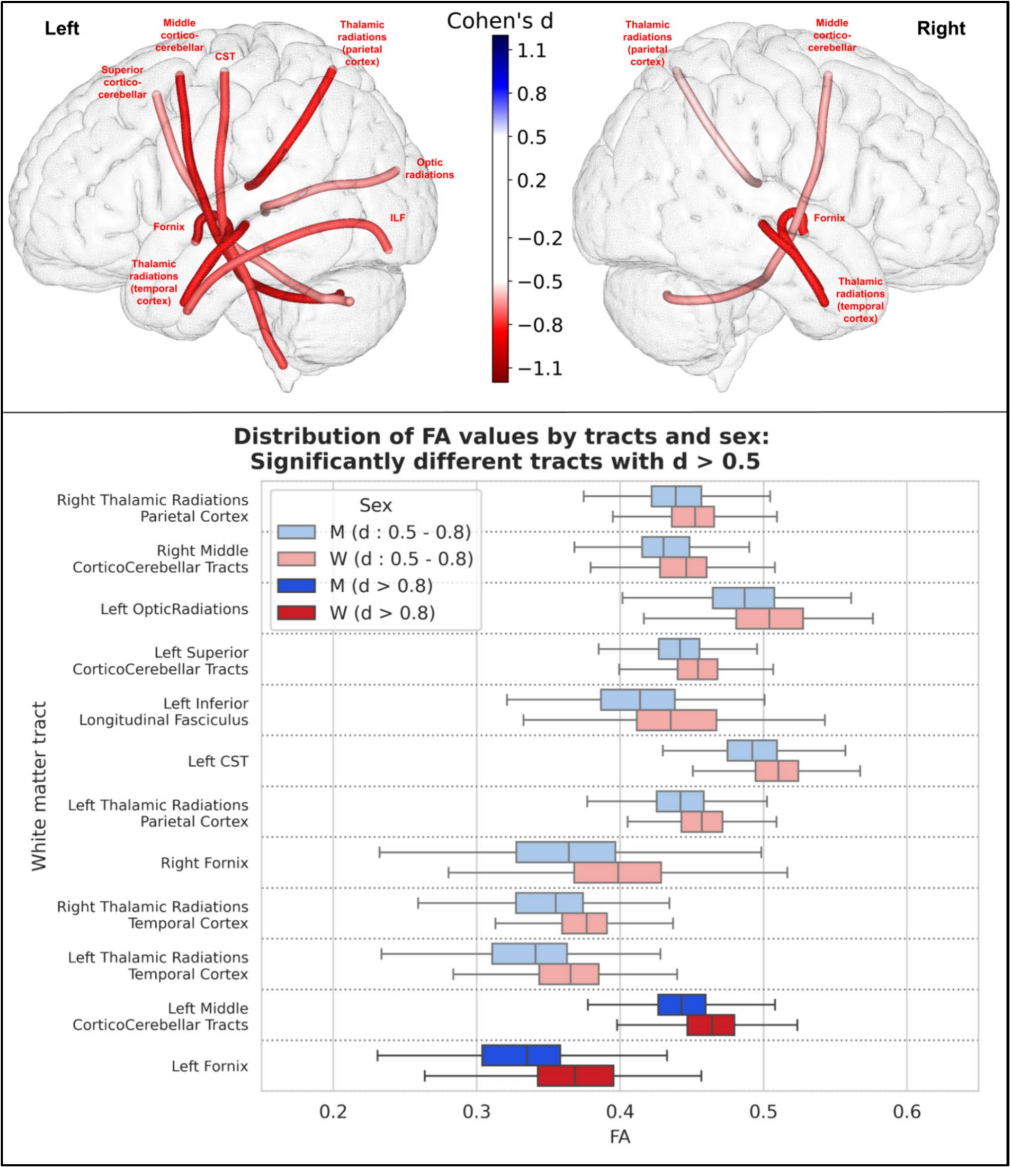
Representation of the 12 white matter tracts with a significant difference in their FA values between men and women and an effect size greater than 0.5. Top: The 12 white matter tracts are represented by their centroid superimposed on a 3D mesh of the brain surface. Left hemisphere tracts are shown on the left side, right hemisphere tracts are shown on the right side, and interhemispheric tracts are shown on both sides. The color represents the direction of the difference (red: greater in women, blue: greater in men), and the color intensity is proportional to the effect size measured by Cohen’s d. Bottom: Distribution of FA values in men (blue) and women (red) of the 12 white matter tracts with a significant difference between men and women and an effect size greater than 0.5, ranked by their Cohen’s d value. Light colors indicate a medium effect size (0.5 < d < 0.8), dark colors indicate a large effect size (d > 0.8)

For fractional anisotropy (FA) estimated from the DTI model, 41 of the 77 tracts were statistically different between men and women (40 showed higher FA in women and 1 showed higher FA in men). Among these, 12 had an effect size greater than 0.5 (all had higher FA in women), and 2 of them had an effect size greater than 0.8: the left fornix, and the left middle cortico-cerebellar tract. The statistical results for these 12 tracts (with a statistically significant difference and a Cohen’s greater than 0.5) are summarized in Fig. 5.

**Fig. 6:**
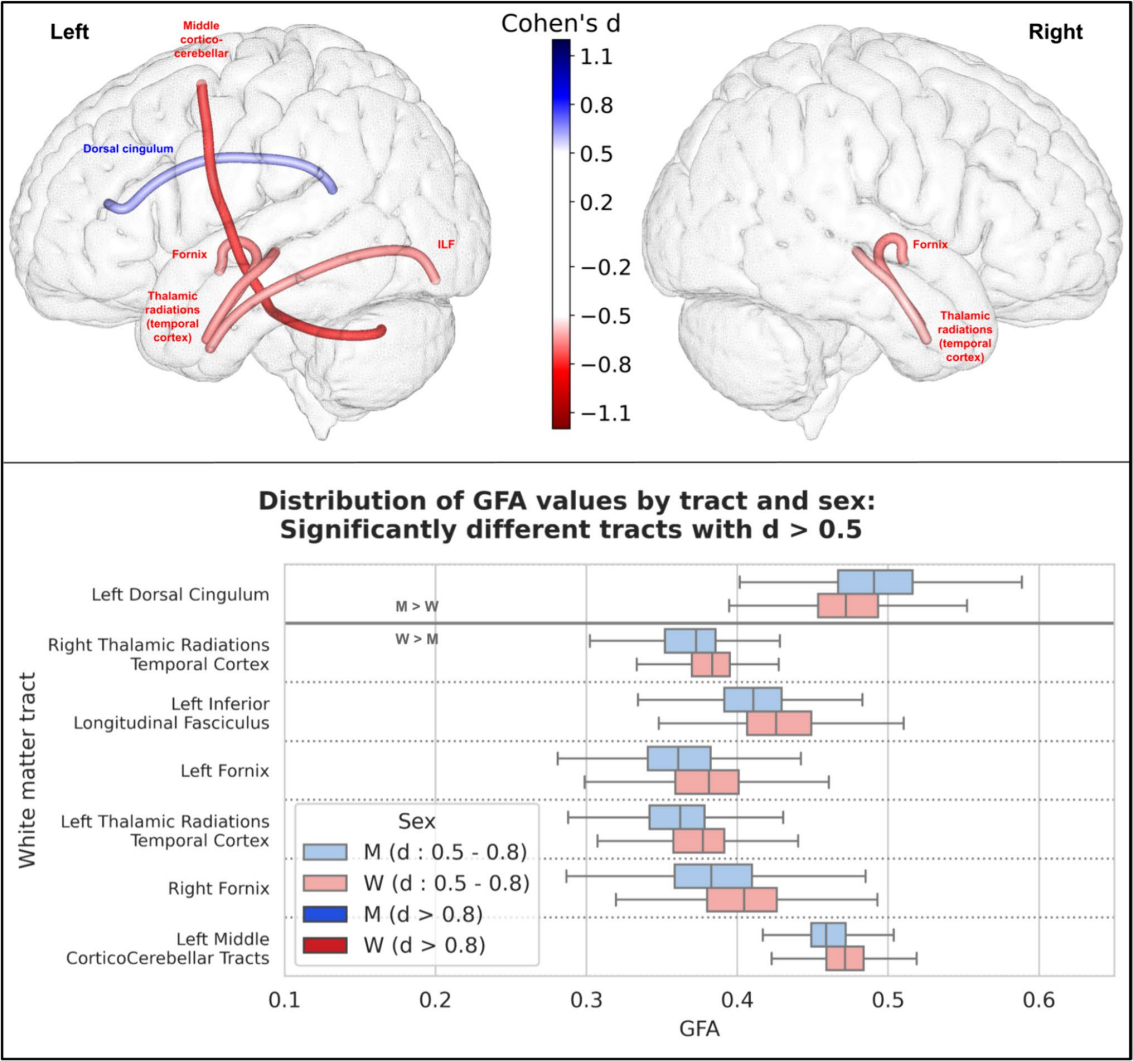
Representation of the 7 white matter tracts with a significant difference in their GFA values between men and women and an effect size greater than 0.5. Top: The 7 white matter tracts are represented by their centroid superimposed on a 3D mesh of the brain surface. Left hemisphere tracts are shown on the left side, right hemisphere tracts are shown on the right side, and interhemispheric tracts are shown on both sides. The color represents the direction of the difference (red: greater in women, blue: greater in men), and the color intensity is proportional to the effect size measured by Cohen’s d. Bottom: Distribution of GFA values in men (blue) and women (red) of the 7 white matter tracts with a significant difference between men and women and an effect size greater than 0.5, ranked by their Cohen’s d value. Light colors indicate a medium effect size (0.5 < d < 0.8), dark colors indicate a large effect size (d > 0.8). GFA was found to be higher in women versus men in all those tracts except for the left dorsal cingulum tract

Generalized fractional anisotropy (GFA) calculated from the Q-ball model yielded similar results, with slightly higher p-values and smaller effect sizes. Thus, 32 tracts were significantly different between men and women, 7 of which had an effect size greater than 0.5 (6 had higher GFA in women and 1 in men), and none greater than 0.8. The results of these 7 tracts are summarized in Fig. 6.

**Fig. 7:**
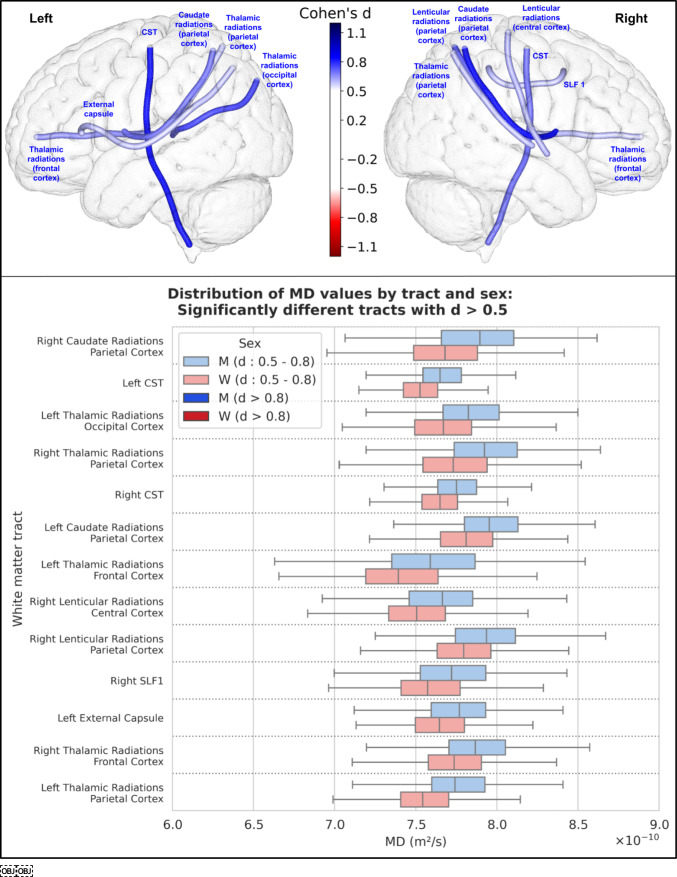
Representation of the 13 white matter tracts with a significant difference in their MD values between men and women and an effect size greater than 0.5. Top: The 13 white matter tracts are represented by their centroid superimposed on a 3D mesh of the brain surface. Left hemisphere tracts are shown on the left side, right hemisphere tracts are shown on the right side, and interhemispheric tracts are shown on both sides. The color represents the direction of the difference (red: greater in women, blue: greater in men), and the color intensity is proportional to the effect size measured by Cohen’s d. Bottom: Distribution of MD values in men (blue) and women (red) of the 13 white matter tracts with a significant difference between men and women and an effect size greater than 0.5, ranked by their Cohen’s d value. Light colors indicate a medium effect size (0.5 < d < 0.8), dark colors indicate a large effect size (d > 0.8)

For mean diffusivity (MD), 34 tracts were significantly different between men and women. 13 had an effect size greater than 0.5 (and none was greater than 0.8), all greater in men. The results of these 13 tracts are shown in Fig. 7.

For both axial and radial diffusivities, the results were similar to those obtained for MD values.

**Fig. 8:**
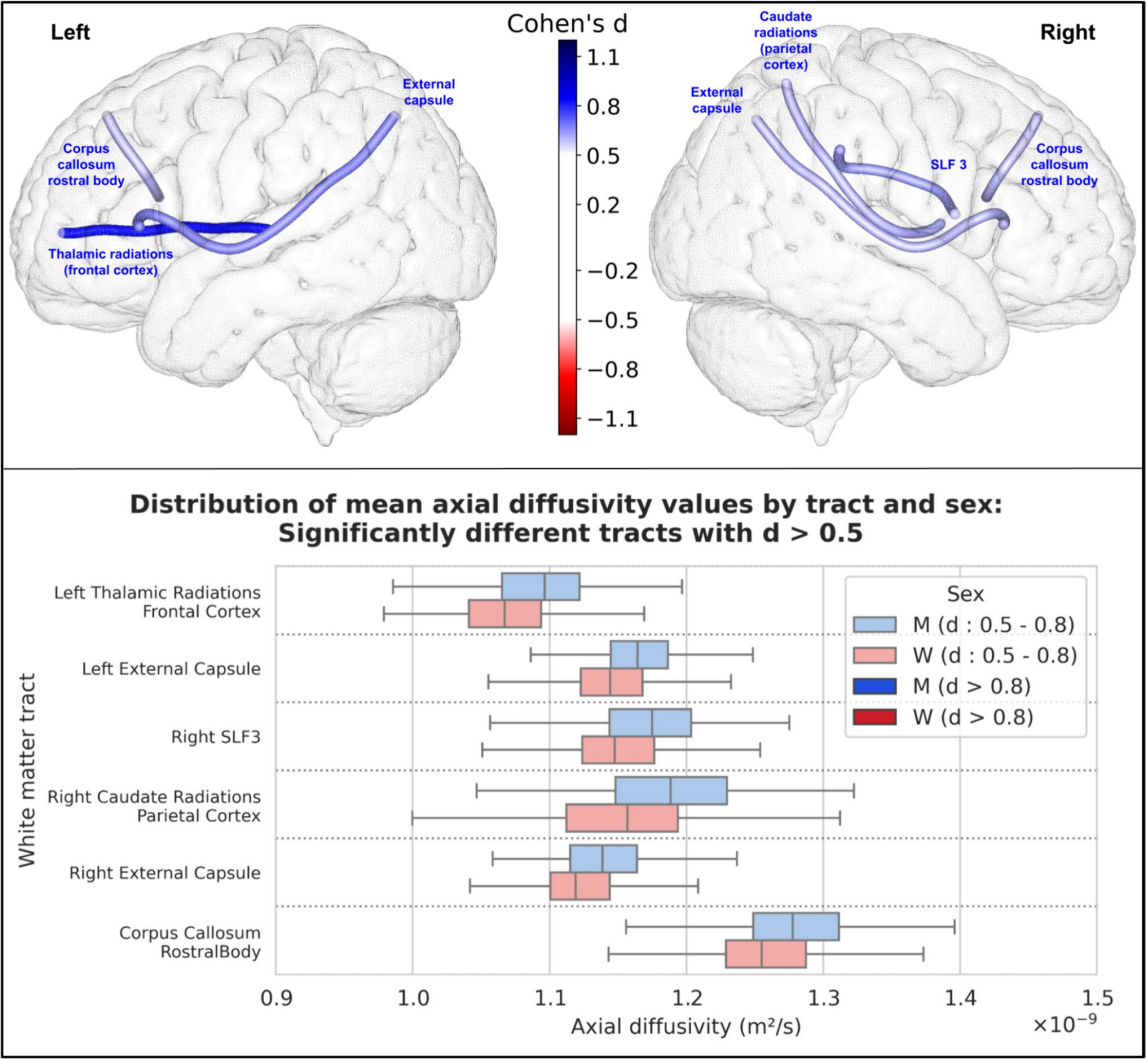
Representation of the 6 white matter tracts with a significant difference in their mean axial diffusivity values between men and women and an effect size greater than 0.5. Top: The 6 white matter tracts are represented by their centroid superimposed on a 3D mesh of the brain surface. Left hemisphere tracts are shown on the left side, right hemisphere tracts are shown on the right side, and interhemispheric tracts are shown on both sides. The color represents the direction of the difference (red: greater in women, blue: greater in men), and the color intensity is proportional to the effect size measured by Cohen’s d. Bottom: Distribution of mean axial diffusivity values in men (blue) and women (red) of the 6 white matter tracts with a significant difference between men and women and an effect size greater than 0.5, ranked by their Cohen’s d value. Light colors indicate a medium effect size (0.5 < d < 0.8), dark colors indicate a large effect size (d > 0.8*)*

For axial diffusivity, 28 tracts were statistically significantly different between men and women. Among these, 6 had an effect size greater than 0.5, and none had an effect size greater than 0.8; all were greater in men. The results of these 6 tracts are shown in Fig. 8.

**Fig. 9:**
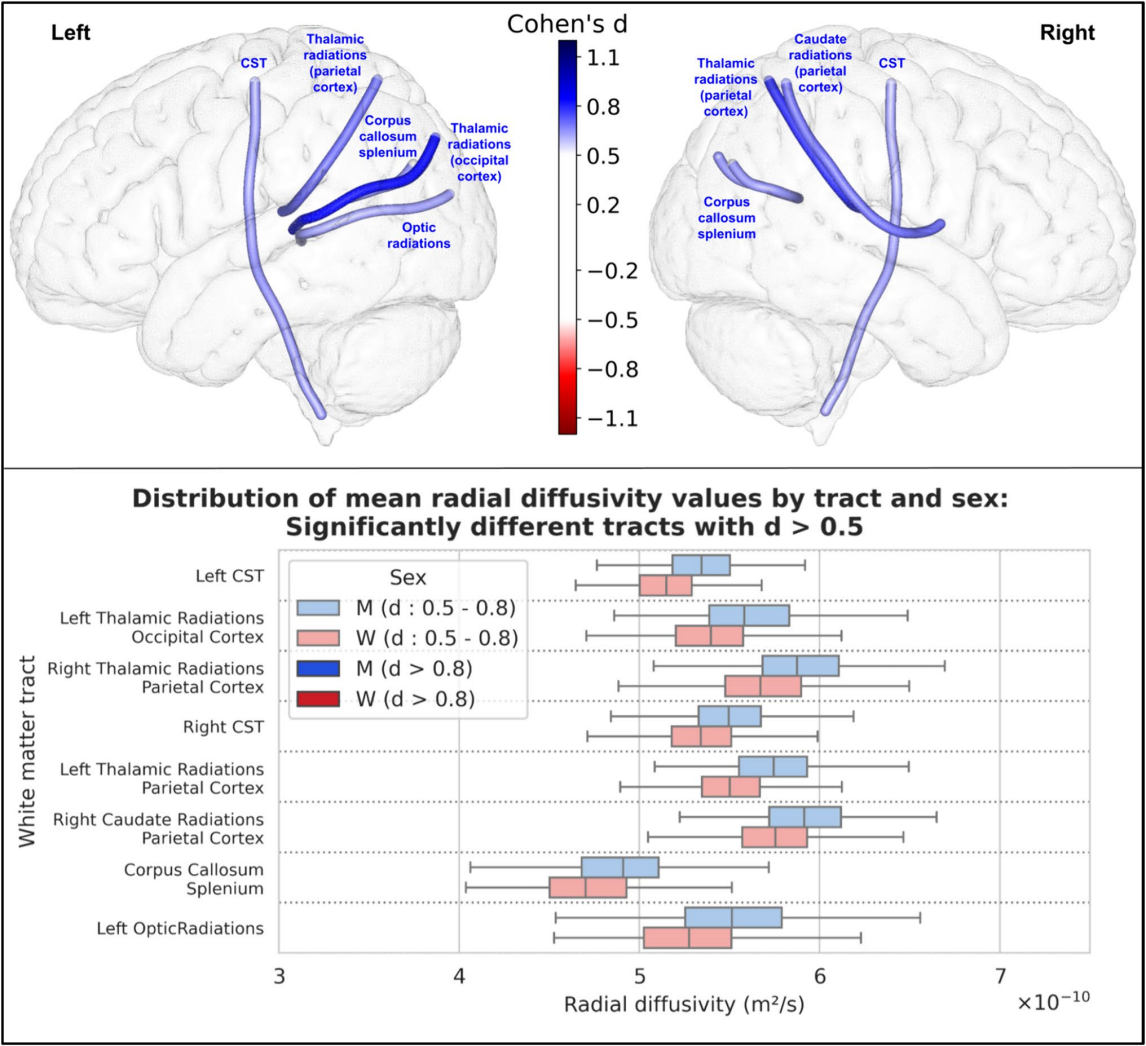
Representation of the 8 white matter tracts with a significant difference in their mean radial diffusivity values between men and women and an effect size greater than 0.5. Top: The 8 white matter tracts are represented by their centroid superimposed on a 3D mesh of the brain surface. Left hemisphere tracts are shown on the left side, right hemisphere tracts are shown on the right side, and interhemispheric tracts are shown on both sides. The color represents the direction of the difference (red: greater in women, blue: greater in men), and the color intensity is proportional to the effect size measured by Cohen’s d. Bottom: Distribution of mean radial diffusivity values in men (blue) and women (red) of the 8 white matter tracts with a significant difference between men and women and an effect size greater than 0.5, ranked by their Cohen’s d value. Light colors indicate a medium effect size (0.5 < d < 0.8), dark colors indicate a large effect size (d > 0.8)

For radial diffusivity, 34 tracts were statistically significantly different between men and women. Among these, 8 had an effect size greater than 0.5, and none had an effect size greater than 0.8. The results of these 8 tracts are shown in Fig. 9.

The NODDI model allowed us to estimate 3 additional microstructural parameters: the neurite density index, the isotropic water volume fraction, and the orientation dispersion index.

**Fig. 10:**
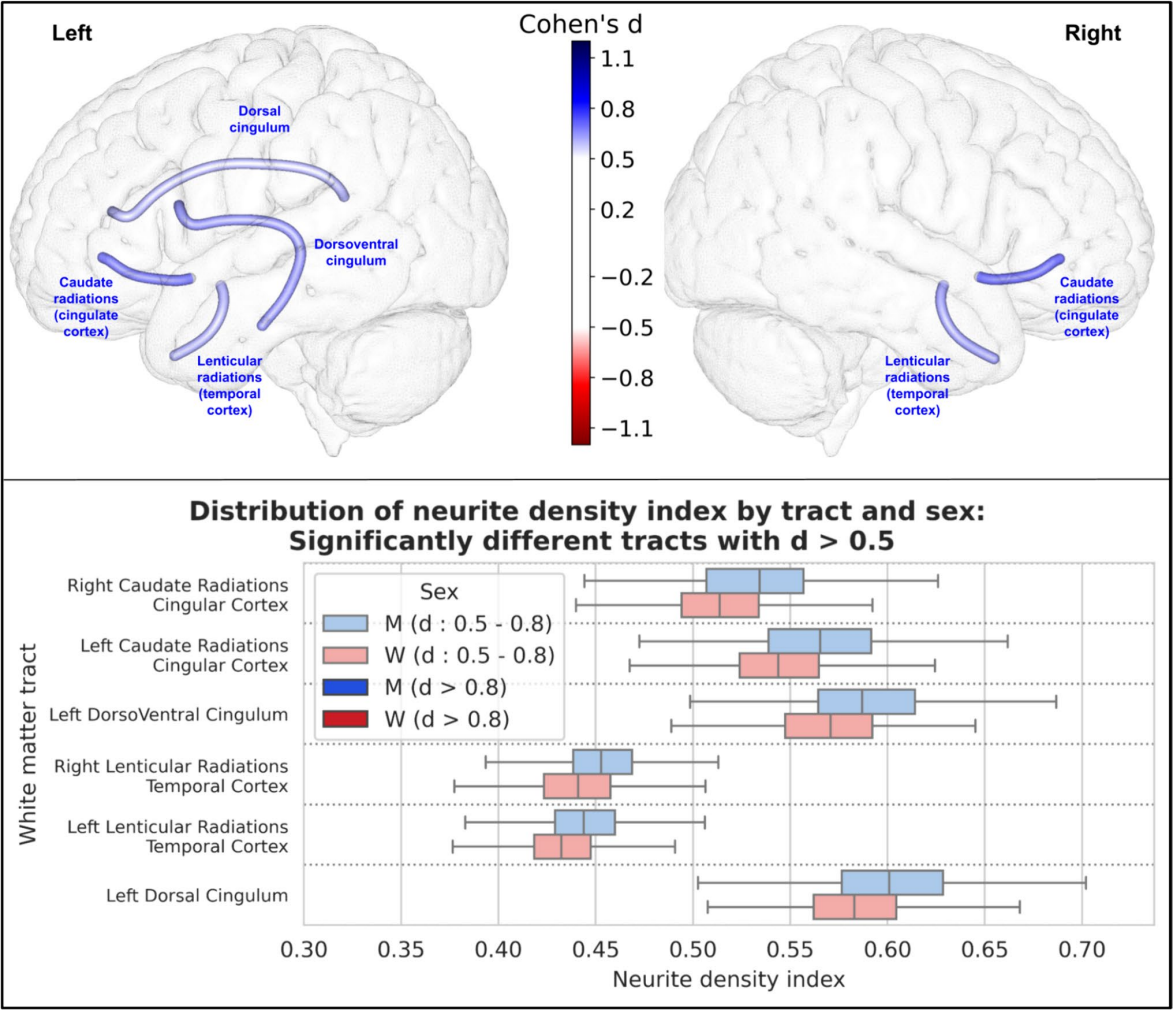
Representation of the 6 white matter tracts with a significant difference in their neurite density index between men and women and an effect size greater than 0.5. Top: The 6 white matter tracts are represented by their centroid superimposed on a 3D mesh of the brain surface. Left hemisphere tracts are shown on the left side, right hemisphere tracts are shown on the right side, and interhemispheric tracts are shown on both sides. The color represents the direction of the difference (red: greater in women, blue: greater in men), and the color intensity is proportional to the effect size measured by Cohen’s d. Bottom: Distribution of neurite density index in men (blue) and women (red) of the 6 white matter tracts with a significant difference between men and women and an effect size greater than 0.5, ranked by their Cohen’s d value. Light colors indicate a medium effect size (0.5 < d < 0.8), dark colors indicate a large effect size (d > 0.8)

For the neurite density index (NDI), 21 tracts were statistically different between men and women. Of these, 6 had an effect size greater than 0.5 (the left dorsal cingulum, the left and right lenticulo-temporal radiations, the left dorso-ventral cingulum, the left cingulo-caudate radiations and the right cingulo-caudate radiations). None had an effect size greater than 0.8. All 6 tracts showed a higher intracellular water fraction in men. The results of these 6 tracts are shown in Fig. 10.

The isotropic water volume fraction showed statistically significant differences between men and women for almost all tracts (76/77). 57 had an effect size greater than 0.5, and 35 of these were greater than 0.8. For all tracts, the isotropic water volume fraction was higher in men. The results are shown in Supplementary Fig. 3.

**Fig. 11:**
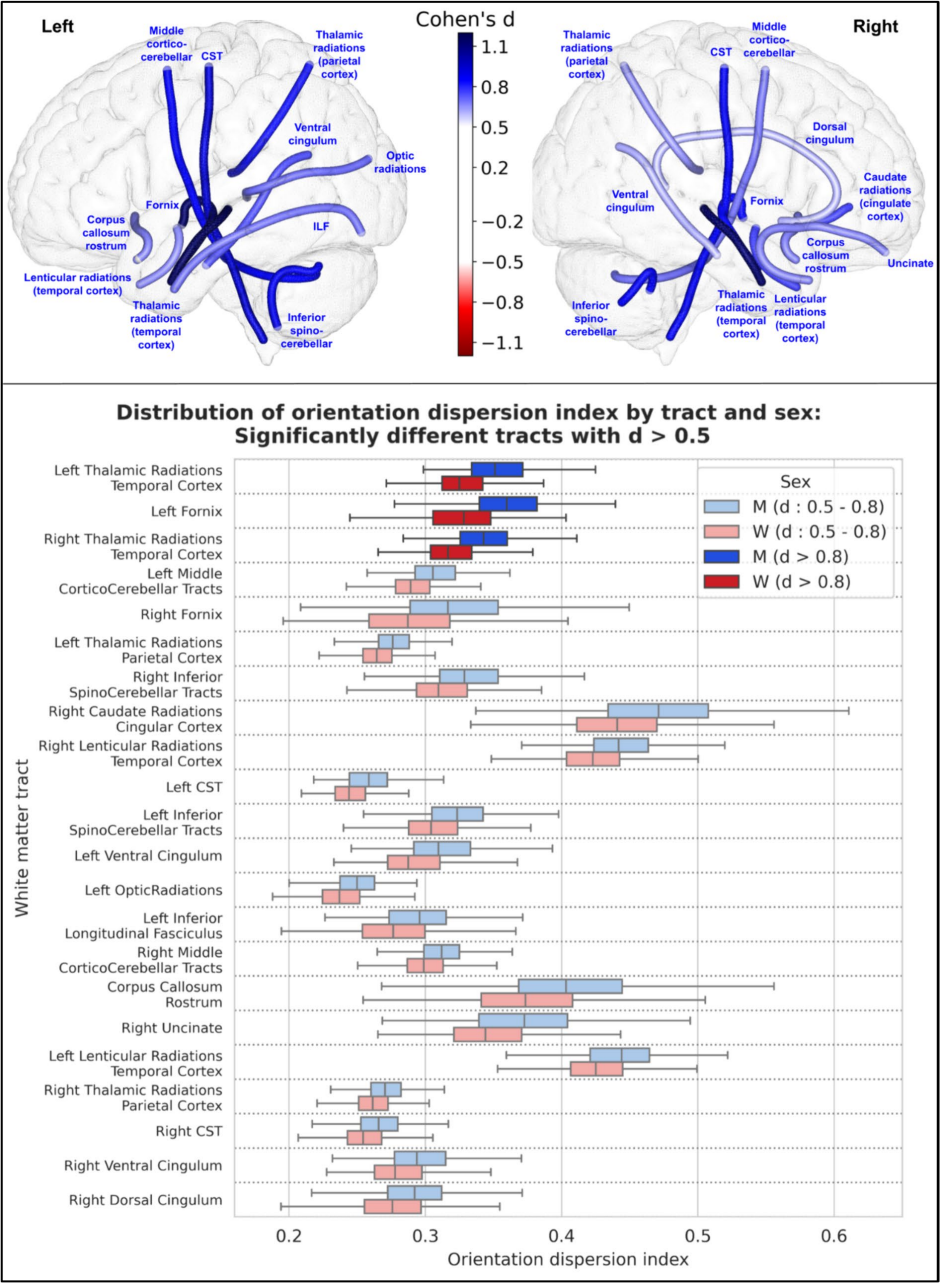
Representation of the 22 white matter tracts with a significant difference in their orientation dispersion index between men and women and an effect size greater than 0.5. Top: The 22 white matter tracts are represented by their centroid superimposed on a 3D mesh of the brain surface. Left hemisphere tracts are shown on the left side, right hemisphere tracts are shown on the right side, and interhemispheric tracts are shown on both sides. The color represents the direction of the difference (red: greater in women, blue: greater in men), and the color intensity is proportional to the effect size measured by Cohen’s d. Bottom: Distribution of orientation dispersion index in men (blue) and women (red) of the 22 white matter tracts with a significant difference between men and women and an effect size greater than 0.5, ranked by their Cohen’s d value. Light colors indicate a medium effect size (0.5 < d < 0.8), dark colors indicate a large effect size (d > 0.8)

For the orientation dispersion index (ODI), 46 tracts showed a statistically significant difference between men and women. 22 of these had an effect size greater than 0.5, and 3 of these had an effect size greater than 0.8: the left fornix, and the left and right thalamo-temporal radiations, all of which were higher in men. The results of these 22 tracts are shown in Fig. 11.

## Discussion

This study provides a detailed and unprecedented overview of white matter tract differences between the sexes in a homogeneous cohort of young healthy adults. While, as expected, there exists a large overlap between men and women across most parameters, including those with the most significant differences, we have identified several robust disparities in white matter tracts between males and females, both in terms of volume and microstructure, some of which exhibit a substantial effect size.

Notably, the tracts displaying the most pronounced differences are those associated with the motor system (such as cortico-spinal tracts and cerebellar tracts) and tracts of the limbic system (including the fornix, cingulum, and tracts connecting the temporal cortex to the basal ganglia, particularly the thalamo-temporal radiations). These findings may be linked to the well-established behavioral distinctions between men and women (Archer 2019) observed in time-limited motor tasks, levels of aggressiveness, and prosocial behavior.

## Volumetric differences

### Total brain volume and white matter volume

We found a significantly higher total brain volume in men, with a relative difference of + 12.6% compared to women. This is a well known and studied fact. Our results are consistent with the meta-analysis by Ruigrok et al. (Ruigrok et al. 2014) who found a relative difference of 12% between men and women and with a more recent study (Ritchie et al. 2018), including included 5216 participants from the UK Biobank, that found a relative difference in total brain volume of + 9.6% in men.

Concerning white matter volume, we also found a greater volume in men (+ 13.6%), which is consistent with the 12.9% relative difference in the meta-analysis by Ruigrok et al. (Ruigrok et al. 2014) and the 12.0% relative difference in the study by Ritchie et al. (Ritchie et al. 2018) in the UK Biobank cohort.

### White matter tract volume

In contrast to global volumetric comparisons, fewer volumetric analyses of white matter tracts have been published in the past. Most white matter analyses have examined regional volumes or focused on a single tract. Since the 1980s, the corpus callosum has been one of the most extensively studied white matter structures. Most studies focusing on it have approximated its volume by measuring its area in a midsagittal section (DeLacoste-Utamsing and Holloway 1982; RichardJ 2005; Ardekani et al. 2013). However, this approach has several limitations, prompting the use of alternative methods to estimate the corpus callosum volume. These methods include voxel-based morphometry (Shiino et al. 2017) or surface-based mesh modeling (Luders et al. 2014), each with its own set of advantages and drawbacks. Another approach, as demonstrated by Pietrasik et al. (Pietrasik et al. 2020) in their study on the volumetric and microstructural aging of the corpus callosum, involves conducting tractography first and then measuring the volume of the entire bundle traversing the corpus callosum (or its subparts). This method offers the advantage of assessing the entire tract rather than just the voxels of the callosal midsagittal area, providing more comprehensive information about the extent of the tract and its hemispheric connections. However, it requires more elaborated dMRI scans to acquire high-resolution diffusion MRI, and is computationally more demanding to reconstruct the corpus callosum more reliably.

In this study, we employed a methodology similar to the latter approach to measure the volume of all major white matter tracts. Therefore, our tract labeled "corpus callosum genu" refers to the entire tract passing through the corpus callosum genu and connecting the left and right frontal cortices.

Because the gray matter/white matter volume ratio also differs between the sexes (Leonard et al. 2008; Jäncke et al. 2014; Luders et al. 2014), we performed normalization to both total brain volume and white matter volume. Although the results were close, the tracts that ended up being significant were not strictly similar using the two normalization methods. In particular, slightly more tracts were statistically significant after normalization to white matter volume (18 tracts versus 16 after normalization to total brain volume). Notably, other sections of the corpus callosum, not just the genu, were significant with this second normalization, but not with the first. However, since normalization to white matter volume seems to increase the differences between the groups, differences that are significant with this method but not after normalization to total brain volume should be interpreted with caution.

While early studies on this topic initially reported a larger corpus callosum volume in women relative to their brain size (DeLacoste-Utamsing and Holloway 1982; RichardJ 2005), subsequent research suggests that much of this difference is attributable to total brain volume (Eliot et al. 2021; Luders et al. 2014). However, this does not contradict the hypothesis that a small proportion of the differences observed in corpus callosum volume may indeed be influenced by sex, as indicated by recent studies where sex explained some variance in its volume (Pietrasik et al. 2020; Potvin et al. 2016). This is further supported by investigations involving men and women matched for identical intracranial volume, which demonstrated slightly greater corpus callosum volume in women (Ardekani et al. 2013; Shiino et al. 2017). In our study, the corpus callosum genu exhibited the most significant difference between men and women in its normalized volume, whether normalized to total brain volume or white matter volume, with strong statistical significance and a medium effect size, indicating greater normalized volume in women. This finding aligns with the aforementioned studies, as well as a connectomic study (Ingalhalikar et al. 2014) which identified greater inter-hemispheric connectivity in women and greater intra-hemispheric connectivity in men. However, subsequent research (Hänggi et al. 2014; Martínez et al. 2017) has tempered these findings, attributing much of the difference in intra- and inter-hemispheric connectivity to brain size rather than gender.

To further explore the association between normalized corpus callosum volume and total brain volume, a linear regression analysis in both sexes was conducted, similar to the approach taken by Leonard et al. (12), and differing slopes between men and women were noticed. An ANCOVA confirmed this association, revealing a strong statistically significant interaction (p = 3.49.10^–65^) between sex and normalized corpus callosum volume after adjusting for total brain volume. Ultimately, while brain volume undoubtedly serves as the primary determinant of normalized corpus callosum volume, we can confidently conclude that sex also exerts an independent effect on it.

One hypothesis to explain these differences is that the greater intra-hemispheric connectivity observed in men promotes fast goal-directed actions, potentially contributing to faster reaction time and higher sensorimotor speed (Gur et al. 2012), as well as the stronger lateralization in men. Conversely, the corpus callosum facilitates interhemispheric communication and enhances bilateral integration of information processed by each hemisphere. This is critical for many high-order cognitive processes that rarely rely on unilateral areas, and may contribute to better performance in certain high-order cognitive functions in women, particularly in areas such as social cognition.

In addition to the corpus callosum genu, 15 other white matter tracts showed a significant difference in normalized volume, but their effect size was smaller than that of the corpus callosum genu, and a greater overlap was found between the two groups. Specifically, we observed that men tended to have larger tracts connecting the frontal areas and the basal ganglia, particularly the lenticular nuclei. These findings may be relevant to certain behavioral differences observed between the sexes, such as higher impulsivity and aggressiveness in men. Contrary to the corpus callosum, the lack of literature on this topic makes it difficult to compare with other results, as only some rare studies, mostly focusing on a single tract (such as the fornix (Cahn et al. 2021) or the anterior commissure (Choi et al. 2011)), included a volumetric analysis. Consequently, reproducibility of these findings would be necessary to confirm their significance.

### Microstructural analysis

In our study, we found a statistically significant difference in FA values (after correction for multiple comparisons) in 41 of the 77 tracts, with an overall higher FA in women (40 of these 41 tracts had higher FA in women, while only one had higher FA in men with a small effect size). This result was consistent for both FA from the traditional DTI model and GFA calculated using the HARDI diffusion solid-angle corrected Q-ball model. Microstructural studies of white matter differences according to sex are heterogeneous and not as numerous as studies of gray matter differences. The parameters measured from the DTI model are the most commonly studied, and among them the most studied is FA. However, the studies reported so far are rather contradictory:

- Consistent with our findings, some cohort studies reported higher overall FA in women (Dunst et al. 2014; Kanaan et al. 2014). In particular, one study was performed in the same HCP cohort as ours, with a focus on the fornix (31), and reported higher total white matter FA in women (and their results regarding the fornix were comparable to ours). A recent meta-analysis (Kochunov et al. 2015) concluded that women had higher overall FA than men, with a relative difference of + 2%.
- Conversely, three studies (17,32,33) conducted on large numbers of subjects (3513, 5216 and 15,628 subjects) from the UKBiobank cohort found an overall higher FA in men in most white matter regions. However, these differences were greatly reduced or eliminated after adjustment for TBV (17), and only few remained significant after this adjustment: higher FA in women in the left inferior longitudinal fasciculus (d = 0.14) and posterior thalamic radiation (d = 0.12); and higher FA in men in the right arcuate fasciculus (d = 0.26), bilateral corticospinal tract (right: d = 0.22, left: d = 0.15), and bilateral superior thalamic radiation (right: d = 0.16, left: d = 0.15).
- Other studies have reported mixed results, with FA values being higher in either men or women depending on the examined tracts (Kanaan et al. 2012, 2014) or voxels across the brain (Hsu et al. 2008; Inano et al. 2011; Chou et al. 2011). In children and adolescents, a recent study performed on 6797 children aged 9–10 years (Lawrence et al. 2023) found regional variations when comparing FA between boys and girls (with some regions demonstrating higher FA in girls and others in boys), and overall higher MD, axial and radial diffusivity in boys, which is consistent with our results in young adults.

The disparities among these results may stem from differences in methodology. Unlike many older studies that measured and compared these parameters in broad brain regions (e.g., assessing the mean fractional anisotropy (FA) of the white matter in the frontal lobe), we computed the mean of these parameters along specific white matter tracts. Our approach focuses on these individual white matter tracts and their associated functions, rather than employing a global regional measure with less specific significance. Additionally, measuring the mean values of these microstructural features along the tract, as we did, is not influenced by tract length or total brain volume (TBV), factors that are crucial for such analyses and were not consistently accounted for in previous studies.

The parameters measured using the DTI model (FA, MD, axial and radial diffusivity) are relevant and have been associated with many important white matter changes during aging (Bennett and Madden 2014) or neuropsychiatric diseases (Pievani et al. 2014). However, DTI has many limitations, and other alternative models have been developed to overcome them. Among them, the NODDI model separately models restricted, hindered, and free water diffusion, which refer to intraneurite, extracellular, and isotropic (free) water components, respectively (Zhang et al. 2012). It thus provides good estimates of some microstructural aspects of the neurites that DTI cannot assess, by measuring neurite density (from the intracellular volume fraction), neurite complexity and fanning (from the orientation density index), or the isotropic water fraction (which, in the brain, is particularly important to consider for areas close to the ventricles or the convexity of the brain, where cerebrospinal fluid, a prototypical isotropic water, may be responsible for partial volume effects in voxels). To our knowledge, the only studies using this model to compare white matter microstructure between males and females were those performed using the UKBiobank cohort (Ritchie et al. 2018; Cox et al. 2016; Lawrence et al. 2021), which reported higher ODI in women for most tracts. Lawrence et al. (39) used another model, the Restriction Spectrum Imaging (RSI) model, to examine white matter microstructure in young healthy subjects (9–10 years) and reported greater NDI in girls.

In our study, among the tracts showing statistically significant differences between men and women using these advanced microstructural parameters, 4 had a large effect size: the left cortico-spinal tract (d = 0.83), the left fornix (d = 0.98), and the left (d = 1.0) and right (d = 0.97) thalamo-temporal radiations. The right cortico-spinal tract and the right fornix were also significantly different between men and women with a slightly smaller effect size, just above the threshold of 0.8 for a large effect size: their Cohen's d was 0.75 and 0.71, respectively. These tracts were among those with the largest differences in all microstructural parameters, as well as other tracts from the motor (cortico-cerebellar tracts) or limbic (cingulum) systems. These microstructural differences in such tracts that are key components of the motor task-based network (cortico-spinal and cortico-cerebellar tracts, as well as tracts connecting the frontal and central cortices to the basal ganglia) and the social cognition network (Wang et al. 2018) (cingulum, fornix, and thalamo-temporal radiations) are particularly interesting because they relate to some of the functions that differ most between men and women (2): time-constrained motor tasks and social interests and skills. The fact that the differences between men and women in these tracts can be found in nearly all diffusion parameters suggests that the underlying differences in white matter structure extend to multiple aspects of microstructure. This highlights the importance of considering microstructure when studying sex differences in the brain, as they can provide deeper insights in the underlying neural mechanisms specific to women and men.

## Limitations of the study

The main limitation of our study is the lack of histological data in the HCP cohort, which prevents a direct comparison between the fiber tracts visualized by Klinger’s dissection (Klinger 1935) or more recent dissection techniques (Zemmoura et al. 2014), and those reconstructed by dMRI tractography. On the other hand, although dissection studies provide detailed anatomical information, they are typically performed in a limited number of subjects, and would likely lack the statistical power to reach significance given the small differences between the two groups. The agreement between our volumetric analysis and published histological data (DeLacoste-Utamsing and Holloway 1982) supports the validity of our method.

The methodology of tractography and fiber tract labeling also has some limitations. In particular, the choice of white matter atlas and the degree of tract subdivision may have some implications when performing tract-based measurements. For example, in the present studies, we analyzed and performed measurements on the inferior longitudinal fasciculus (ILF) as a whole, whereas other studies (Latini et al. 2017) have divided this tract into three components: the fusiform, the lingual, and the dorsolateral-occipital ILF. Performing measurements of microstructural parameters on these subdivisions rather than on the entire tract may yield different results because it is possible that, for a given microstructural parameter, only one subdivision of the tract differs between men and women, potentially underestimating the sex effect in some tracts investigated as a whole. However, this increases our confidence in the differences we actually identified, because such a hypothesis would lead to an underestimation, not an overestimation, of the difference between the groups.

The possibility of a partial volume effect, especially for tracts near the ventricles, is also a limitation of the method used. The cerebro-spinal fluid compartment and the ventricle volume are larger in men than in women (Ruigrok et al. 2014). This means the partial volume effect is stronger in men, which might affect some of the microstructural parameters. The NODDI model takes this into account by modeling the isotropic water fraction, which we indeed found to be higher in men. The other two compartments (intracellular and extracellular water fraction) are thus not affected, and neither are the computed ODI and NDI. However, parameters computed using the DTI and Q-ball models are susceptible to partial volume effects. Due to their anatomical location near the lateral ventricles, the fornices and cingulum are the tracts most susceptible to this effect. We found no differences in these tracts in MD, axial and radial diffusivities. However, we found that women had higher FA and GFA values in both fornices, while men had higher GFA values in the dorsal cingulum. A greater partial volume effect in men would make anisotropy seem lower in men, leading to an overestimation of the difference found in the fornices and an underestimation of the difference found in the dorsal cingulum. A study that specifically focused on the diffusion parameters of the fornix (Cahn et al. 2021) in a subset of subjects from the HCP cohort found results that were comparable to those observed in our study. The authors acknowledged that, even at the relatively high (1.25 mm isotropic) spatial resolution of the HCP cohort, partial volume effect had indeed an influence on the results. Therefore, it is important to consider that the differences observed in FA and GFA values in the fornices may be slightly overestimated.

Another limitation is that the HCP cohort is quite homogeneous, consisting of healthy adults between 22 and 35 years old. Therefore, our results cannot be generalized to other age groups, especially to the elderly. Age-related changes in white matter microstructure may influence the observed sex differences, as has been reported in different age groups (Lawrence et al. 2021; Toschi et al. 2020), with earlier aging of white matter microstructural parameters in men than in women.

It is noteworthy that the measured differences between the sexes remained small in our study, with substantial overlap in the parameter distributions. This small effect size may explain why other studies, especially those with smaller sample sizes, may find different results. We emphasize the importance of working with large datasets to perform such analyses, as was done here and in similar studies of the UKBiobank cohort (Ritchie et al. 2018; Cox et al. 2016; Lawrence et al. 2021). In the future, meta-analyses that aggregate the results of similar studies conducted in different cohorts of subjects of different ages and origins, with the necessary correction for site effects, may help to resolve remaining inconsistencies in the field.

Finally, it is important to mention the issue of reproducibility and comparability between studies. The other studies on the same topic used different acquisition protocols and diffusion processing pipelines, and possibly measured different parameters. The present study was performed on the HCP cohort, whose diffusion protocol was refined during the first two years of the HCP project to achieve a standardized and state-of-the-art acquisition protocol for high angular resolution diffusion imaging (Sotiropoulos et al. 2013). Since then, the HCP cohort has inspired numerous "HCP-style" studies using a similar acquisition protocol, thus facilitating the comparison of their results. In addition, harmonization methods to account for inter- and intra-site variability (Pinto et al. 2020; Fortin et al. 2017) have been developed in recent years to improve the comparability of the technique. However, reproducibility on topics such as tractography algorithm (Schilling et al. 2019) or white matter tract segmentation (Rheault et al. 2020, 2022a, 2022b) is still imperfect, and the development and use of standardized diffusion preprocessing and analysis methods should be an important goal to facilitate comparability between studies (Tax et al. 2022 Apr).

## Conclusion

Our study has demonstrated that while there are numerous similarities in white matter tracts and structural connectivity between men and women, there are also discernible differences related to sex. These disparities were strongly significant in certain white matter tract volumes, even after normalization to total brain volume, as well as in microstructural parameters, and demonstrated medium to high effect size. The tracts exhibiting the most differences were tracts from motor (cortico-spinal tracts, cortico-cerebellar tracts) or limbic (cingulum, fornix, thalamo-temporal radiations) systems. Future research can expand upon our findings to delve deeper into the intricate relationship between brain connectivity and the cognitive and behavioral traits that exhibit differences between men and women.

## Supplementary Information

Below is the link to the electronic supplementary material.

Supplementary file1 (DOCX 8 KB)

Supplementary file2 (PNG 349 KB)

Supplementary file3 (PNG 256 KB)

Supplementary file4 (PNG 1474 KB)

Supplementary file5 (DOCX 19 KB)

## Data Availability

Data were provided by the Human Connectome Project, WU-Minn Consortium (Principal Investigators: David Van Essen and Kamil Ugurbil; 1U54MH091657) funded by the 16 NIH Institutes and Centers that support the NIH Blueprint for Neuroscience Research; and by the McDonnell Center for Systems Neuroscience at Washington University. Diffusion MRI data processing was performed using the Ginkgo toolbox developed by the CEA/NeuroSpin team, freely available online at https://framagit.org/cpoupon/gkg
