# Supplementary material for: Sex-related variability of white matter tracts in the whole HCP cohort
Source: Brain Struct Funct. 2024 Jul 16;229(7):1713–35. doi: 10.1007/s00429-024-02833-0 (PMC11374878; doi:10.1007/s00429-024-02833-0)
Supplement: Supplementary file 1 — Supplementary file1 (DOCX 8 KB) [file 429_2024_2833_MOESM1_ESM.docx]

***Deep white matter atlas constitution***

The white matter atlas was constructed using all 1065 subjects from the HCP cohort. It was performed by first performing a dimensionality reduction on each subject’s tractogram, using an intra-subject clustering algorithm in order to reduce the number of fiber, then an inter-subject fiber clustering at the level of the entire HCP cohort in order to reliably extract the deep white matter tract.

The dimensionality reduction step used the intra-subject clustering algorithm described by Guevara et al. (60), which groups streamlines together according to their geometric properties. Fibers are first clustered into 4 different regions (left hemisphere, right hemisphere, interhemispheric, and cerebellum). Fibers from each region are then grouped by length range (10 length groups), and for each resulting group, a fiber density map is computed from the voxels crossed by the fibers. Each density map is then finely parcellated using a k-means algorithm, and a connectivity matrix is computed from the reconstructed white matter fibers to determine the structural connectivity profile of each pair of parcels. A lower threshold is applied to discard pairs of parcels with low connectivity (< 1 %). Finally, an average-link hierarchical clustering algorithm is applied to the connectivity matrix to extract clusters of connected parcels. The resulting parcel clusters are then used to identify white matter fiber clusters corresponding to fibers that intersect the parcel clusters for at least 60 % of their length, representing groups of fibers (also called fascicles) of similar length that strongly connect adjacent voxels. A final watershed step is performed to differentiate fiber clusters according to their extremities. To further reduce the representation of the entire set of fiber clusters obtained at an individual scale, each fiber cluster (or fascicle) is represented by its centroid, which corresponds to the fiber that represents the shortest distance to all other fibers belonging to the cluster. This last step results in a centroid map that provides a sparse (and efficient) representation of all fascicles at an individual scale.

After intra-subject clustering were performed on the 1065 subjects, a cross-subject fiber clustering algorithm was applied to all the individual cluster centroid maps registered in the MNI template, in order to generate maps of fascicles common to the population, using the HDBscan algorithm (61) with the following parameters (optimized by a grid search to maximize the number of clusters obtained): normalization factor 6, neighbor count 5, minimum cluster size 10, minimum subject percentage 2.5 %.

From the results of this cross-subject fiber clustering, deep white matter tracts were independently identified by two trained neuroanatomists using manual ROI selection based on the neuroanatomical literature. All resulting tracts were manually curated to remove residual artifactual fibers. This allowed the construction of a deep white matter fiber atlas from the entire HCP cohort, containing 77 tracts: 15 association tracts for each hemisphere, 19 projection tracts for each hemisphere, 8 interhemispheric tracts, and 1 intracerebellar tract. The anatomical T1 volume was used to segment the corpus callosum into 7 parts according to Witelson's segmentation (62), and these ROIs were used to divide the corpus callosum fibers into the same 7 parts. Projection fibers connecting the cortex and the basal ganglia were segmented according to the cortical areas they connect (i.e., central, cingulate, frontal, parietal, occipital, temporal, and insular). Cortico-cerebellar tracts were segmented according to the cerebellar peduncle through which they pass.

The entire diffusion analysis pipeline is shown in Supplementary Figure 1. An overview of the resulting atlas is shown in Supplementary Figure 2.
