# Supplementary figures and images for: Sex-related variability of white matter tracts in the whole HCP cohort
Source: Brain Struct Funct. 2024 Jul 16;229(7):1713–35. doi: 10.1007/s00429-024-02833-0 (PMC11374878; doi:10.1007/s00429-024-02833-0)

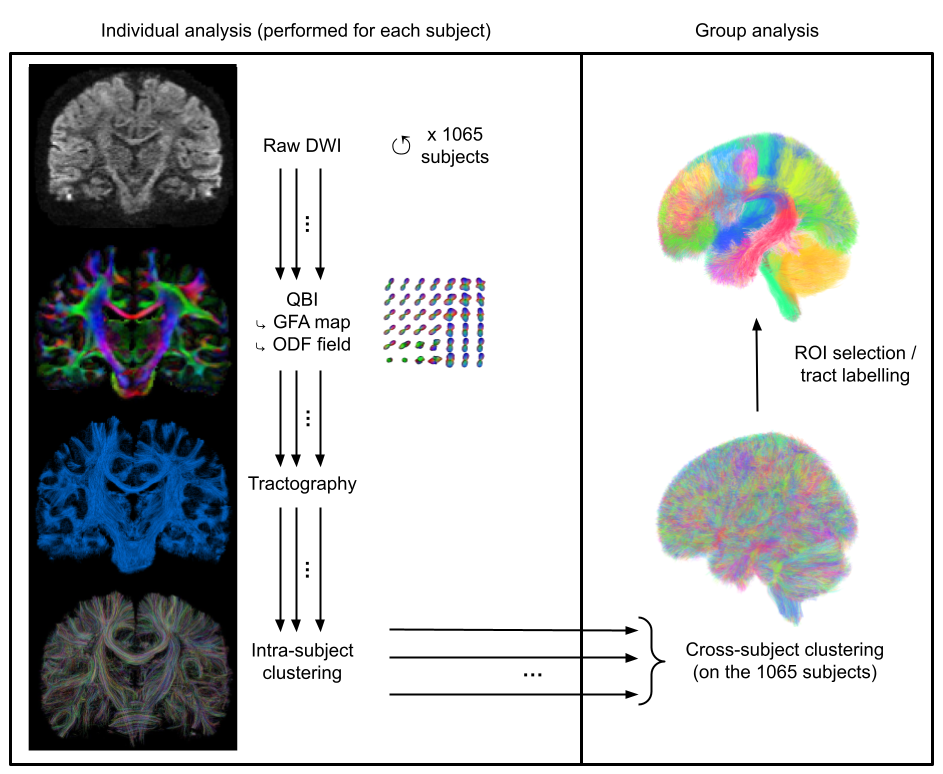

Supplement: Supplementary file 2 — Supplementary file2 (PNG 349 KB) [file 429_2024_2833_MOESM2_ESM.png]

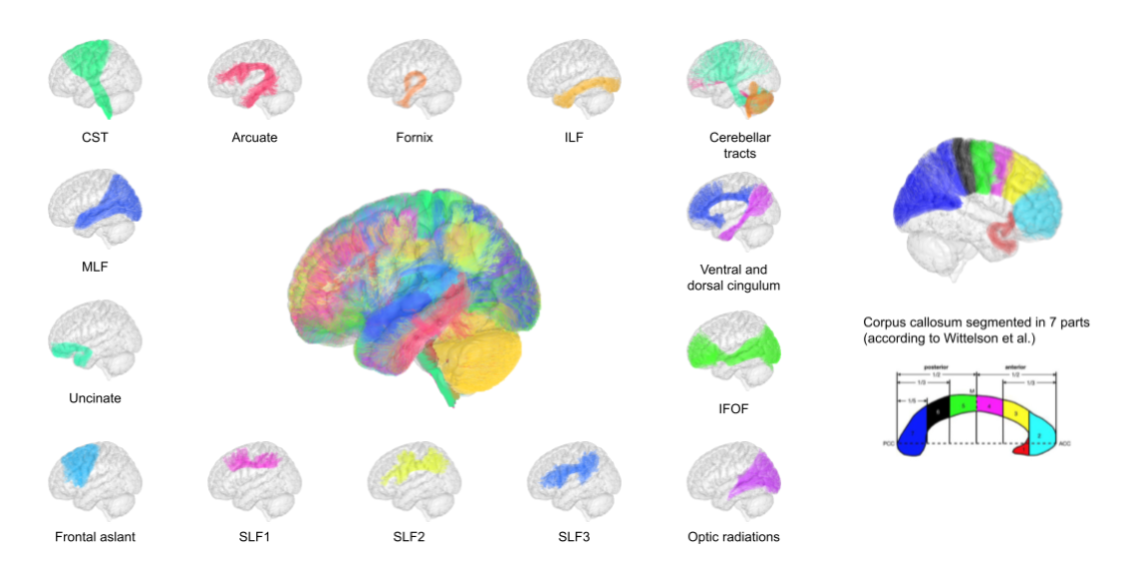

Supplement: Supplementary file 3 — Supplementary file3 (PNG 256 KB) [file 429_2024_2833_MOESM3_ESM.png]

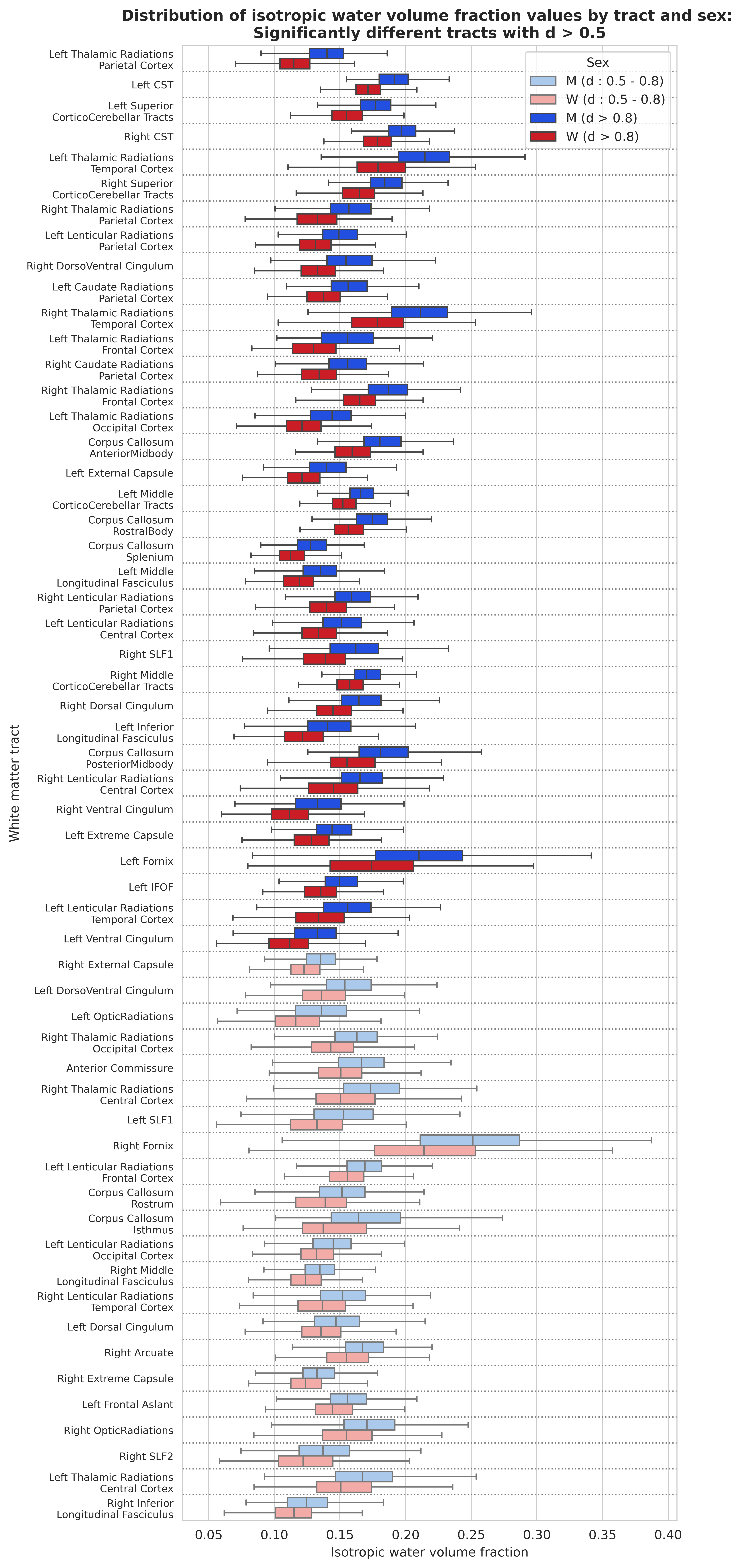

Supplement: Supplementary file 4 — Supplementary file4 (PNG 1474 KB) [file 429_2024_2833_MOESM4_ESM.png]
