# Supplementary material for: Sex-related variability of white matter tracts in the whole HCP cohort
Source: Brain Struct Funct. 2024 Jul 16;229(7):1713–35. doi: 10.1007/s00429-024-02833-0 (PMC11374878; doi:10.1007/s00429-024-02833-0)
Supplement: Supplementary file 5 — Supplementary file5 (DOCX 19 KB) [file 429_2024_2833_MOESM5_ESM.docx]

| **Tract** | **FA** | **GFA** | **MD** | **Axial diffusivity** | **Radial diffusivity** | **Neurite density index** | **Isotropic water volume fraction** | **Orientation dispersion index** |
| --- | --- | --- | --- | --- | --- | --- | --- | --- |
| Anterior_Commissure | -6.1 %; p = 2.26.10^-11^; d = -0.42 | -4.0 %; p = 4.44.10^-09^; d = -0.37 | NS | NS | NS | NS | 11.8 %; p = 8.91.10^-31^; d = 0.74 | 4.4 %; p = 3.36.10^-13^; d = 0.46 |
| CorpusCallosum_AnteriorMidbody | NS | NS | 2.5 %; p = 2.78.10^-11^; d = 0.41 | 1.3 %; p = 1.75.10^-07^; d = 0.32 | 3.8 %; p = 7.62.10^-10^; d = 0.38 | NS | 11.9 %; p = 8.55.10^-38^; d = 0.82 | NS |
| CorpusCallosum_Genu | NS | NS | NS | NS | NS | NS | NS | NS |
| CorpusCallosum_Isthmus | -2.4 %; p = 3.23.10^-05^; d = -0.26 | NS | 3.0 %; p = 2.34.10^-05^; d = 0.26 | NS | 4.8 %; p = 2.01.10^-05^; d = 0.26 | NS | 13.8 %; p = 3.27.10^-22^; d = 0.61 | NS |
| CorpusCallosum_PosteriorMidbody | -2.4 %; p = 2.87.10^-09^; d = -0.37 | NS | 3.0 %; p = 1.37.10^-12^; d = 0.44 | 1.4 %; p = 4.68.10^-09^; d = 0.36 | 5.0 %; p = 1.28.10^-11^; d = 0.42 | NS | 12.5 %; p = 3.40.10^-33^; d = 0.76 | NS |
| CorpusCallosum_RostralBody | NS | NS | 2.1 %; p = 2.59.10^-13^; d = 0.45 | 1.9 %; p = 7.27.10^-16^; d = 0.5 | 2.5 %; p = 3.59.10^-07^; d = 0.31 | NS | 10.4 %; p = 1.31.10^-41^; d = 0.86 | NS |
| CorpusCallosum_Rostrum | -7.0 %; p = 4.88.10^-11^; d = -0.41 | NS | NS | NS | NS | NS | 10.0 %; p = 1.26.10^-18^; d = 0.55 | 5.8 %; p = 4.88.10^-17^; d = 0.52 |
| CorpusCallosum_Splenium | -2.4 %; p = 1.73.10^-14^; d = -0.48 | -1.4 %; p = 1.67.10^-06^; d = -0.3 | 1.6 %; p = 3.90.10^-11^; d = 0.41 | NS | 3.9 %; p = 3.10.10^-18^; d = 0.54 | NS | 12.4 %; p = 3.13.10^-60^; d = 1.07 | NS |
| Left_Arcuate | NS | NS | NS | NS | NS | NS | 9.5 %; p = 5.58.10^-17^; d = 0.52 | NS |
| Left_CST | -3.3 %; p = 8.21.10^-25^; d = -0.65 | -1.5 %; p = 1.99.10^-11^; d = -0.42 | 1.7 %; p = 1.52.10^-28^; d = 0.7 | NS | 3.5 %; p = 9.79.10^-36^; d = 0.79 | 0.9 %; p = 2.04.10^-06^; d = 0.29 | 10.3 %; p = 2.85.10^-83^; d = 1.3 | 4.9 %; p = 6.60.10^-24^; d = 0.63 |
| Left_Caudate_Radiations_Central_Cortex | -3.0 %; p = 4.67.10^-06^; d = -0.28 | NS | 2.6 %; p = 3.51.10^-07^; d = 0.31 | NS | 3.5 %; p = 6.28.10^-08^; d = 0.33 | -1.9 %; p = 4.74.10^-05^; d = -0.25 | 10.0 %; p = 5.63.10^-12^; d = 0.43 | NS |
| Left_Caudate_Radiations_Cingular_Cortex | NS | 2.5 %; p = 2.75.10^-10^; d = 0.39 | NS | -1.4 %; p = 1.36.10^-06^; d = -0.3 | NS | 1.9 %; p = 3.96.10^-06^; d = 0.28 | 7.4 %; p = 1.13.10^-08^; d = 0.35 | 3.1 %; p = 9.57.10^-13^; d = 0.44 |
| Left_Caudate_Radiations_Frontal_Cortex | NS | 1.5 %; p = 1.56.10^-05^; d = 0.27 | NS | NS | NS | NS | 4.2 %; p = 1.03.10^-07^; d = 0.33 | NS |
| Left_Caudate_Radiations_Parietal_Cortex | NS | NS | 1.8 %; p = 8.66.10^-21^; d = 0.59 | 1.8 %; p = 3.50.10^-13^; d = 0.45 | 1.8 %; p = 4.25.10^-09^; d = 0.36 | NS | 12.9 %; p = 1.95.10^-57^; d = 1.04 | NS |
| Left_Cingulum_Long |  |  |  |  |  |  |  |  |
| Left_Dorsal_Cingulum | 2.5 %; p = 2.94.10^-08^; d = 0.34 | 3.6 %; p = 1.07.10^-17^; d = 0.53 | NS | 1.5 %; p = 5.04.10^-09^; d = 0.36 | NS | 3.8 %; p = 8.45.10^-23^; d = 0.62 | 13.3 %; p = 1.13.10^-36^; d = 0.81 | -3.8 %; p = 4.37.10^-08^; d = -0.34 |
| Left_External_Capsule | NS | 2.7 %; p = 3.57.10^-09^; d = 0.37 | NS | NS | NS | 2.8 %; p = 2.32.10^-15^; d = 0.5 | 13.7 %; p = 1.30.10^-42^; d = 0.89 | NS |
| Left_Extreme_Capsule | NS | NS | 1.6 %; p = 2.13.10^-16^; d = 0.51 | 1.8 %; p = 7.03.10^-20^; d = 0.57 | 1.4 %; p = 9.43.10^-07^; d = 0.3 | NS | 14.5 %; p = 3.96.10^-52^; d = 0.98 | NS |
| Left_Fornix | NS | NS | 1.6 %; p = 3.41.10^-15^; d = 0.49 | 1.0 %; p = 4.33.10^-06^; d = 0.28 | 2.3 %; p = 2.35.10^-13^; d = 0.45 | NS | 12.3 %; p = 1.92.10^-37^; d = 0.82 | NS |
| Left_Frontal_Aslant | -10.4 %; p = 1.68.10^-41^; d = -0.87 | -4.9 %; p = 2.61.10^-19^; d = -0.56 | NS | NS | NS | NS | 18.3 %; p = 1.50.10^-41^; d = 0.86 | 9.1 %; p = 4.68.10^-49^; d = 0.96 |
| Left_IFOF | NS | NS | NS | NS | NS | NS | 7.4 %; p = 5.22.10^-15^; d = 0.49 | NS |
| Left_Inferior_Longitudinal_Fasciculus | -2.5 %; p = 6.90.10^-11^; d = -0.41 | -1.8 %; p = 6.47.10^-08^; d = -0.34 | NS | NS | NS | NS | 11.0 %; p = 3.30.10^-42^; d = 0.88 | NS |
| Left_Inferior_SpinoCerebellar_Tracts | -3.4 %; p = 2.96.10^-08^; d = -0.34 | NS | NS | -2.2 %; p = 7.13.10^-14^; d = -0.47 | NS | 1.9 %; p = 6.32.10^-12^; d = 0.43 | NS | 5.6 %; p = 9.18.10^-23^; d = 0.62 |
| Left_Lenticular_Radiations_Central_Cortex | -5.0 %; p = 8.28.10^-15^; d = -0.48 | -2.5 %; p = 9.63.10^-09^; d = -0.35 | 45.6 %; p = 2.48.10^-13^; d = 0.45 | 45.1 %; p = 2.62.10^-13^; d = 0.45 | 45.9 %; p = 2.41.10^-13^; d = 0.45 | NS | 7.7 %; p = 4.52.10^-16^; d = 0.5 | 9.0 %; p = 5.24.10^-39^; d = 0.83 |
| Left_Lenticular_Radiations_Frontal_Cortex | NS | NS | 1.8 %; p = 2.45.10^-09^; d = 0.36 | 1.6 %; p = 2.48.10^-06^; d = 0.29 | NS | NS | 11.5 %; p = 4.75.10^-41^; d = 0.87 | NS |
| Left_Lenticular_Radiations_Occipital_Cortex | NS | NS | NS | NS | NS | NS | 8.6 %; p = 9.92.10^-27^; d = 0.68 | NS |
| Left_Lenticular_Radiations_Parietal_Cortex | -2.2 %; p = 1.49.10^-08^; d = -0.35 | -1.5 %; p = 1.65.10^-06^; d = -0.3 | NS | NS | 2.4 %; p = 7.72.10^-08^; d = 0.33 | NS | 8.8 %; p = 1.48.10^-23^; d = 0.63 | NS |
| Left_Lenticular_Radiations_Temporal_Cortex | NS | NS | NS | NS | NS | 1.3 %; p = 2.72.10^-05^; d = 0.26 | 13.7 %; p = 1.82.10^-63^; d = 1.11 | NS |
| Left_Middle_CorticoCerebellar_Tracts | NS | 1.5 %; p = 1.11.10^-05^; d = 0.27 | NS | NS | NS | 1.6 %; p = 3.69.10^-06^; d = 0.28 | 10.1 %; p = 1.56.10^-24^; d = 0.64 | 2.6 %; p = 2.79.10^-15^; d = 0.49 |
| Left_Middle_Longitudinal_Fasciculus | -4.7 %; p = 4.96.10^-37^; d = -0.81 | -2.5 %; p = 5.96.10^-24^; d = -0.64 | NS | NS | NS | NS | 8.5 %; p = 2.17.10^-55^; d = 1.02 | 4.8 %; p = 3.12.10^-31^; d = 0.74 |
| Left_OpticRadiations | -2.3 %; p = 4.05.10^-08^; d = -0.34 | -1.9 %; p = 9.48.10^-08^; d = -0.33 | NS | NS | NS | NS | 13.6 %; p = 6.69.10^-50^; d = 0.96 | NS |
| Left_SLF1 | -3.7 %; p = 2.21.10^-18^; d = -0.55 | -2.2 %; p = 3.28.10^-09^; d = -0.37 | 2.6 %; p = 2.10.10^-12^; d = 0.43 | NS | 4.8 %; p = 7.14.10^-18^; d = 0.53 | NS | 14.4 %; p = 6.89.10^-37^; d = 0.81 | 3.1 %; p = 7.22.10^-11^; d = 0.41 |
| Left_SLF2 | NS | 2.6 %; p = 8.98.10^-09^; d = 0.35 | NS | 1.6 %; p = 4.89.10^-08^; d = 0.34 | NS | 3.1 %; p = 6.74.10^-11^; d = 0.4 | 16.7 %; p = 4.14.10^-30^; d = 0.72 | NS |
| Left_SLF3 | NS | NS | NS | NS | NS | NS | 8.2 %; p = 1.60.10^-09^; d = 0.37 | NS |
| Left_Superior_CorticoCerebellar_Tracts | NS | NS | 1.5 %; p = 2.34.10^-06^; d = 0.29 | 1.0 %; p = 2.66.10^-06^; d = 0.29 | 2.0 %; p = 4.84.10^-05^; d = 0.25 | NS | 9.3 %; p = 1.63.10^-13^; d = 0.46 | NS |
| Left_Thalamic_Radiations_Central_Cortex | -2.8 %; p = 4.65.10^-19^; d = -0.56 | -1.4 %; p = 2.13.10^-09^; d = -0.37 | NS | NS | NS | NS | 12.7 %; p = 5.82.10^-86^; d = 1.32 | 2.7 %; p = 9.39.10^-15^; d = 0.48 |
| Left_Thalamic_Radiations_Frontal_Cortex | -2.7 %; p = 2.39.10^-06^; d = -0.29 | NS | 1.7 %; p = 3.12.10^-05^; d = 0.26 | NS | 2.8 %; p = 1.04.10^-07^; d = 0.33 | -1.5 %; p = 4.18.10^-05^; d = -0.25 | 9.9 %; p = 5.42.10^-20^; d = 0.58 | 2.9 %; p = 3.65.10^-05^; d = 0.26 |
| Left_Thalamic_Radiations_Occipital_Cortex | NS | NS | 2.5 %; p = 3.11.10^-19^; d = 0.56 | 2.5 %; p = 3.63.10^-30^; d = 0.72 | 2.4 %; p = 2.59.10^-10^; d = 0.39 | NS | 15.7 %; p = 3.12.10^-56^; d = 1.03 | NS |
| Left_Thalamic_Radiations_Parietal_Cortex | -3.0 %; p = 1.06.10^-14^; d = -0.48 | -1.7 %; p = 8.04.10^-08^; d = -0.33 | 2.3 %; p = 2.61.10^-24^; d = 0.64 | NS | 4.0 %; p = 3.88.10^-28^; d = 0.69 | NS | 15.4 %; p = 4.09.10^-58^; d = 1.05 | 2.9 %; p = 5.42.10^-08^; d = 0.34 |
| Left_Thalamic_Radiations_Temporal_Cortex | -3.5 %; p = 2.72.10^-28^; d = -0.7 | -1.7 %; p = 1.79.10^-11^; d = -0.42 | 3.0 %; p = 1.11.10^-16^; d = 0.5 | 1.3 %; p = 3.16.10^-06^; d = 0.28 | 4.8 %; p = 2.12.10^-21^; d = 0.58 | NS | 17.6 %; p = 1.63.10^-99^; d = 1.45 | 3.4 %; p = 1.39.10^-21^; d = 0.6 |
| Left_Uncinate | -8.0 %; p = 2.28.10^-34^; d = -0.78 | -3.9 %; p = 6.96.10^-19^; d = -0.56 | NS | NS | NS | NS | 15.8 %; p = 1.04.10^-69^; d = 1.16 | 6.6 %; p = 1.57.10^-49^; d = 0.95 |
| Left_Ventral_Cingulum | -3.0 %; p = 1.68.10^-05^; d = -0.27 | NS | NS | NS | NS | 2.3 %; p = 1.51.10^-09^; d = 0.37 | 7.8 %; p = 2.49.10^-13^; d = 0.46 | 3.2 %; p = 1.15.10^-08^; d = 0.35 |
| ParallelFibers | -4.1 %; p = 7.14.10^-13^; d = -0.45 | NS | NS | NS | NS | 1.3 %; p = 4.31.10^-05^; d = 0.25 | 15.8 %; p = 7.95.10^-48^; d = 0.93 | 5.3 %; p = 2.45.10^-19^; d = 0.56 |
| Right_Arcuate | NS | 2.2 %; p = 4.88.10^-09^; d = 0.36 | NS | NS | NS | 8.6 %; p = 3.82.10^-67^; d = 1.16 | 10.9 %; p = 8.93.10^-23^; d = 0.61 | 8.3 %; p = 3.28.10^-72^; d = 1.2 |
| Right_CST | -2.3 %; p = 3.62.10^-08^; d = -0.34 | -1.0 %; p = 6.99.10^-06^; d = -0.28 | 1.4 %; p = 1.42.10^-21^; d = 0.6 | NS | 2.8 %; p = 1.75.10^-22^; d = 0.61 | 1.0 %; p = 3.21.10^-07^; d = 0.32 | 9.8 %; p = 6.70.10^-78^; d = 1.25 | 4.2 %; p = 6.10.10^-18^; d = 0.54 |
| Right_Caudate_Radiations_Central_Cortex | -3.9 %; p = 2.11.10^-25^; d = -0.66 | -2.1 %; p = 5.54.10^-17^; d = -0.52 | 36.3 %; p = 1.08.10^-17^; d = 0.53 | 35.7 %; p = 1.15.10^-17^; d = 0.53 | 36.6 %; p = 1.05.10^-17^; d = 0.53 | NS | 10.9 %; p = 3.54.10^-67^; d = 1.14 | 5.4 %; p = 6.34.10^-28^; d = 0.69 |
| Right_Caudate_Radiations_Cingular_Cortex | NS | NS | 2.8 %; p = 1.41.10^-07^; d = 0.33 | 2.3 %; p = 2.69.10^-07^; d = 0.32 | 3.3 %; p = 1.62.10^-06^; d = 0.3 | NS | 11.9 %; p = 4.74.10^-17^; d = 0.52 | NS |
| Right_Caudate_Radiations_Frontal_Cortex | -3.9 %; p = 1.44.10^-06^; d = -0.3 | NS | NS | -2.2 %; p = 8.51.10^-10^; d = -0.38 | NS | 2.3 %; p = 3.03.10^-09^; d = 0.36 | 7.4 %; p = 8.99.10^-09^; d = 0.35 | 4.2 %; p = 1.55.10^-21^; d = 0.6 |
| Right_Caudate_Radiations_Parietal_Cortex | -2.7 %; p = 3.66.10^-08^; d = -0.34 | NS | NS | NS | 2.3 %; p = 2.25.10^-07^; d = 0.32 | NS | 4.9 %; p = 3.31.10^-12^; d = 0.43 | 2.1 %; p = 3.33.10^-06^; d = 0.29 |
| Right_Cingulum_Long | NS | NS | 2.7 %; p = 5.70.10^-29^; d = 0.71 | 2.6 %; p = 7.07.10^-17^; d = 0.52 | 2.8 %; p = 1.17.10^-20^; d = 0.58 | NS | 14.0 %; p = 1.60.10^-56^; d = 1.03 | NS |
| Right_Dorsal_Cingulum |  |  |  |  |  |  |  |  |
| Right_External_Capsule | NS | NS | 1.3 %; p = 5.08.10^-10^; d = 0.38 | NS | 1.9 %; p = 4.49.10^-08^; d = 0.34 | 1.9 %; p = 5.75.10^-08^; d = 0.34 | 13.6 %; p = 6.15.10^-49^; d = 0.95 | NS |
| Right_Extreme_Capsule | -2.6 %; p = 3.89.10^-06^; d = -0.29 | NS | NS | NS | NS | NS | 14.7 %; p = 6.43.10^-59^; d = 1.06 | 4.1 %; p = 2.29.10^-11^; d = 0.42 |
| Right_Fornix | NS | 1.1 %; p = 3.40.10^-05^; d = 0.26 | 1.2 %; p = 4.40.10^-11^; d = 0.41 | 1.6 %; p = 1.57.10^-16^; d = 0.51 | NS | NS | 10.4 %; p = 7.99.10^-40^; d = 0.84 | NS |
| Right_Frontal_Aslant | NS | NS | NS | NS | NS | 1.4 %; p = 2.60.10^-06^; d = 0.29 | 7.8 %; p = 7.80.10^-20^; d = 0.57 | NS |
| Right_IFOF | -9.8 %; p = 1.08.10^-28^; d = -0.71 | -5.4 %; p = 3.53.10^-19^; d = -0.57 | NS | NS | NS | NS | 13.8 %; p = 1.05.10^-24^; d = 0.66 | 8.9 %; p = 2.02.10^-25^; d = 0.66 |
| Right_Inferior_Longitudinal_Fasciculus | NS | NS | 1.2 %; p = 2.61.10^-07^; d = 0.32 | 0.8 %; p = 3.32.10^-05^; d = 0.26 | 1.6 %; p = 1.93.10^-06^; d = 0.3 | NS | 6.4 %; p = 2.55.10^-13^; d = 0.46 | NS |
| Right_Inferior_SpinoCerebellar_Tracts | -5.1 %; p = 4.58.10^-15^; d = -0.49 | -2.2 %; p = 1.13.10^-07^; d = -0.33 | NS | -1.8 %; p = 2.30.10^-08^; d = -0.35 | NS | 1.2 %; p = 6.33.10^-05^; d = 0.25 | 9.8 %; p = 6.70.10^-78^; d = 1.25 | 5.9 %; p = 4.08.10^-25^; d = 0.65 |
| Right_Lenticular_Radiations_Central_Cortex | -3.1 %; p = 4.53.10^-09^; d = -0.36 | -1.7 %; p = 5.85.10^-05^; d = -0.25 | NS | NS | NS | NS | 9.1 %; p = 2.73.10^-19^; d = 0.56 | 3.7 %; p = 2.12.10^-10^; d = 0.39 |
| Right_Lenticular_Radiations_Frontal_Cortex | -6.4 %; p = 3.37.10^-21^; d = -0.59 | -3.5 %; p = 1.22.10^-14^; d = -0.48 | 39.8 %; p = 4.34.10^-10^; d = 0.38 | 39.3 %; p = 4.63.10^-10^; d = 0.38 | 40.1 %; p = 4.21.10^-10^; d = 0.38 | NS | 6.9 %; p = 2.13.10^-12^; d = 0.44 | 7.7 %; p = 3.00.10^-30^; d = 0.72 |
| Right_Lenticular_Radiations_Occipital_Cortex | NS | NS | 1.9 %; p = 1.07.10^-16^; d = 0.53 | 1.9 %; p = 1.57.10^-07^; d = 0.33 | 1.9 %; p = 5.76.10^-10^; d = 0.39 | NS | 13.3 %; p = 1.56.10^-41^; d = 0.89 | NS |
| Right_Lenticular_Radiations_Parietal_Cortex | NS | NS | NS | NS | NS | 1.5 %; p = 1.44.10^-05^; d = 0.27 | 4.1 %; p = 2.10.10^-05^; d = 0.26 | NS |
| Right_Lenticular_Radiations_Temporal_Cortex | -2.3 %; p = 2.09.10^-10^; d = -0.39 | -1.5 %; p = 3.06.10^-06^; d = -0.29 | NS | NS | 2.2 %; p = 1.71.10^-09^; d = 0.37 | NS | 7.5 %; p = 3.32.10^-16^; d = 0.51 | NS |
| Right_Middle_CorticoCerebellar_Tracts | NS | NS | 1.7 %; p = 6.20.10^-17^; d = 0.52 | 1.3 %; p = 7.85.10^-08^; d = 0.33 | 2.1 %; p = 7.85.10^-14^; d = 0.46 | NS | 12.9 %; p = 4.28.10^-48^; d = 0.94 | NS |
| Right_Middle_Longitudinal_Fasciculus | -2.5 %; p = 6.21.10^-05^; d = -0.25 | NS | NS | NS | NS | 2.0 %; p = 4.97.10^-08^; d = 0.34 | 8.2 %; p = 3.39.10^-16^; d = 0.51 | 3.1 %; p = 5.96.10^-22^; d = 0.6 |
| Right_OpticRadiations | -3.1 %; p = 7.28.10^-18^; d = -0.54 | -1.1 %; p = 4.53.10^-06^; d = -0.28 | NS | NS | NS | 1.5 %; p = 2.46.10^-09^; d = 0.37 | 8.5 %; p = 1.76.10^-51^; d = 0.98 | 4.1 %; p = 5.18.10^-26^; d = 0.67 |
| Right_SLF1 | NS | NS | 1.2 %; p = 4.46.10^-06^; d = 0.28 | NS | 2.0 %; p = 1.45.10^-07^; d = 0.32 | NS | 8.5 %; p = 2.60.10^-25^; d = 0.65 | NS |
| Right_SLF2 | NS | NS | NS | NS | NS | NS | 10.0 %; p = 1.87.10^-22^; d = 0.61 | NS |
| Right_SLF3 | -2.0 %; p = 1.64.10^-05^; d = -0.27 | NS | 1.9 %; p = 3.05.10^-16^; d = 0.51 | 0.9 %; p = 2.85.10^-05^; d = 0.26 | 2.8 %; p = 3.04.10^-14^; d = 0.47 | NS | 16.0 %; p = 1.10.10^-39^; d = 0.84 | NS |
| Right_Superior_CorticoCerebellar_Tracts | NS | NS | 1.5 %; p = 6.69.10^-08^; d = 0.34 | 1.6 %; p = 2.27.10^-11^; d = 0.42 | NS | NS | 11.3 %; p = 2.34.10^-19^; d = 0.57 | -2.7 %; p = 2.99.10^-09^; d = -0.37 |
| Right_Thalamic_Radiations_Central_Cortex | NS | NS | 1.9 %; p = 1.34.10^-11^; d = 0.42 | 2.0 %; p = 9.93.10^-18^; d = 0.54 | 1.9 %; p = 5.70.10^-06^; d = 0.28 | NS | 9.6 %; p = 5.14.10^-18^; d = 0.54 | -2.5 %; p = 4.06.10^-10^; d = -0.39 |
| Right_Thalamic_Radiations_Frontal_Cortex | -1.8 %; p = 7.33.10^-08^; d = -0.33 | NS | NS | NS | NS | NS | 10.9 %; p = 2.45.10^-66^; d = 1.14 | 2.4 %; p = 2.36.10^-11^; d = 0.42 |
| Right_Thalamic_Radiations_Occipital_Cortex | -2.9 %; p = 2.91.10^-07^; d = -0.32 | NS | 2.6 %; p = 1.51.10^-09^; d = 0.38 | NS | 3.7 %; p = 6.83.10^-10^; d = 0.38 | -2.1 %; p = 2.11.10^-08^; d = -0.35 | 12.5 %; p = 6.89.10^-27^; d = 0.68 | NS |
| Right_Thalamic_Radiations_Parietal_Cortex | NS | NS | 1.6 %; p = 1.72.10^-15^; d = 0.5 | 1.2 %; p = 3.42.10^-12^; d = 0.43 | 2.0 %; p = 8.44.10^-12^; d = 0.42 | NS | 11.1 %; p = 7.50.10^-56^; d = 1.02 | 1.9 %; p = 2.33.10^-09^; d = 0.37 |
| Right_Thalamic_Radiations_Temporal_Cortex | NS | NS | 1.7 %; p = 2.67.10^-11^; d = 0.41 | NS | 2.6 %; p = 8.71.10^-11^; d = 0.4 | NS | 12.0 %; p = 1.46.10^-31^; d = 0.74 | NS |
| Right_Uncinate | -2.7 %; p = 1.16.10^-15^; d = -0.5 | -1.2 %; p = 2.74.10^-06^; d = -0.29 | 2.4 %; p = 4.03.10^-23^; d = 0.62 | 1.1 %; p = 7.37.10^-09^; d = 0.36 | 3.6 %; p = 7.45.10^-25^; d = 0.65 | NS | 15.3 %; p = 1.06.10^-61^; d = 1.09 | 3.0 %; p = 4.93.10^-16^; d = 0.51 |
| Right_Ventral_Cingulum | -7.1 %; p = 7.21.10^-34^; d = -0.76 | -3.2 %; p = 3.12.10^-16^; d = -0.51 | NS | NS | NS | NS | 14.8 %; p = 3.17.10^-58^; d = 1.05 | 6.6 %; p = 8.95.10^-53^; d = 0.98 |

*Supplementary table 1. Complete results of comparisons between men and women for all tracts and all microstructural parameters: FA, GFA, MD, axial diffusivity, radial diffusivity, neurite density index, isotropic water volume fraction and orientation dispersion index. For each tract, if a significant difference was found, the percentage difference between the sexes is shown with the associated p-value and Cohen's d. If the comparisons were not significant, "NS" is shown.*
